# Cdk5 phosphorylation-induced SIRT2 nuclear translocation promotes the death of dopaminergic neurons in Parkinson’s disease

**DOI:** 10.1038/s41531-022-00311-0

**Published:** 2022-04-20

**Authors:** Jianguo Yan, Pei Zhang, Jie Tan, Mao Li, Xingfeng Xu, Xiaoyun Shao, Fang Fang, Zhenyou Zou, Yali Zhou, Bo Tian

**Affiliations:** 1 Department of Physiology, Faculty of Basic Medical Science, Guilin Medical University, 1 Zhiyuan Road, Guilin, Guangxi Province 541199 P. R. China; 2 Guangxi Key Laboratory of Brain and Cognitive Neuroscience, Guilin Medical University, 1 Zhiyuan Road, Guilin, Guangxi Province 541199 P. R. China; 3 Department of Neurobiology, Tongji Medical School, Huazhong University of Science and Technology, 13 Hangkong Road, Wuhan, Hubei Province 430030 P. R. China; 4 Department of Microbiology, Faculty of Basic Medical Science, Guilin Medical University, 1 Zhiyuan Road, Guilin, Guangxi Province 541199 P. R. China

**Keywords:** Parkinson's disease, Cellular neuroscience

## Abstract

NAD-dependent protein deacetylase Sirtuin 2 (SIRT2), which regulates several cellular pathways by deacetylating multiple substrates, has been extensively studied in the context of Parkinson’s disease (PD). Although several studies based on the MPTP model of PD show that SIRT2 deletion can protect against dopaminergic neuron loss, the precise mechanisms of SIRT2-mediated neuronal death have largely remained unknown. Here, we show that SIRT2 knockout can effectively ameliorate anomalous behavioral phenotypes in transgenic mouse models of PD. Importantly, in both cellular and animal models of PD, it was observed that SIRT2 translocates from the cytoplasm to the nucleus. Further, the nuclear translocation of SIRT2 promotes neuronal death. Moreover, the cyclin-dependent kinase 5 (Cdk5)-mediated phosphorylation of SIRT2 at the Ser331 and Ser335 sites appears to be necessary for such nuclear translocation. Taken together, the results provide insights into the mechanisms involved in the regulation of neuronal death during PD progression via the Cdk5-dependent nuclear–cytoplasmic shuttling of SIRT2.

## Introduction

Parkinson’s disease (PD), a secondary degenerative disease of the central nervous system (CNS), is characterized by the progressive loss of dopaminergic (DA) neurons in the substantia nigra pars compacta (SNpc). The signs and symptoms of PD are caused by high levels of DA neuronal death in the SNpc and include tremors, bradykinesia, rigid muscles, speech and motor impairments, postural and balance disabilities, and difficulties in automatic movements^1^. Although PD has been studied extensively for almost 200 years, the precise mechanism underlying DA neuronal death remains largely unclear.

Recently, several studies have demonstrated the link between neurodegenerative diseases and histone deacetylases (HDACs)^2^. Evidence from *Drosophila* PD models developed using ectopic expression of human α-synuclein suggests that histone deacetylase 6 (HDAC6) plays a critical role in protecting DA neurons and preventing the development of α-synuclein inclusions^3^. Meanwhile, studies have also reported that under oxidative stress, the intracellular trafficking of HDAC4 makes cells expressing pathogenic α-synuclein mutants vulnerable^4^. Furthermore, a series of pharmacological studies have demonstrated that HDAC inhibitors can provide neuroprotection, preventing DA neuronal death in PD^5,6^. Sirtuins, also called silent information regulator 2 (Sir2) proteins, are members of the class III HDAC family. Sirtuins show distinctive subcellular localization and play a crucial role in neurodegeneration^7^. Mammalian SIRT family proteins (SIRT1–7) have been implicated in the regulation of multiple cellular processes, including inflammation, the cell cycle, DNA repair, apoptosis, stress resistance, and energy metabolism^8–10^. Among the seven Sirtuins, SIRT2 is primarily found in the cytoplasm; SIRT1, SIRT6, and SIRT7 are predominantly expressed in the nucleus; and SIRT3–5 are mainly present in the mitochondria^11^. The contribution of SIRT1 to PD pathophysiology is being increasingly understood. A recent study revealed that SIRT1 deacetylates heat shock factor 1 (HSF1) and increases HSP70 RNA and protein levels to protect against α-synuclein aggregation, which forming insoluble fibrils in pathological conditions characterized by Lewy bodies in PD^12^. Resveratrol, an activator of SIRT1, was found to significantly delay neuronal loss in a mouse model of PD^13^. More importantly, accumulating evidence indicates that SIRT2 could play an opposing role to SIRT1 in neurodegenerative diseases^14^. However, little is known about the exact mechanisms underlying SIRT2 involvement in PD pathophysiology.

In this study, an MPTP-induced SIRT2 knockout (KO) mouse model and α-synuclein-A30P*A53T transgenic mouse model lacking SIRT2 were used to examine the protective role of SIRT2 deletion in PD. Then, studies were performed in a series of PD models, including environmental and genetic models, to determine whether the expression and subcellular localization of SIRT2 change in SNpc neurons. Furthermore, the pro-death effects of SIRT2 nuclear localization were characterized by overexpressing nuclear localization signal (NLS)-SIRT2 in neurons. Finally, cyclin-dependent kinase 5 (Cdk5), a critical protein kinase expressed in mature neurons, was found to be necessary for the nuclear–cytoplasmic shuttling of SIRT2 via its direct phosphorylation at the Ser331 and Ser335 sites.

## Results

### SIRT2 deletion ameliorates behavioral phenotypes and DA neuron loss in MPTP-induced and transgenic mouse models of PD

**Fig. 1:**
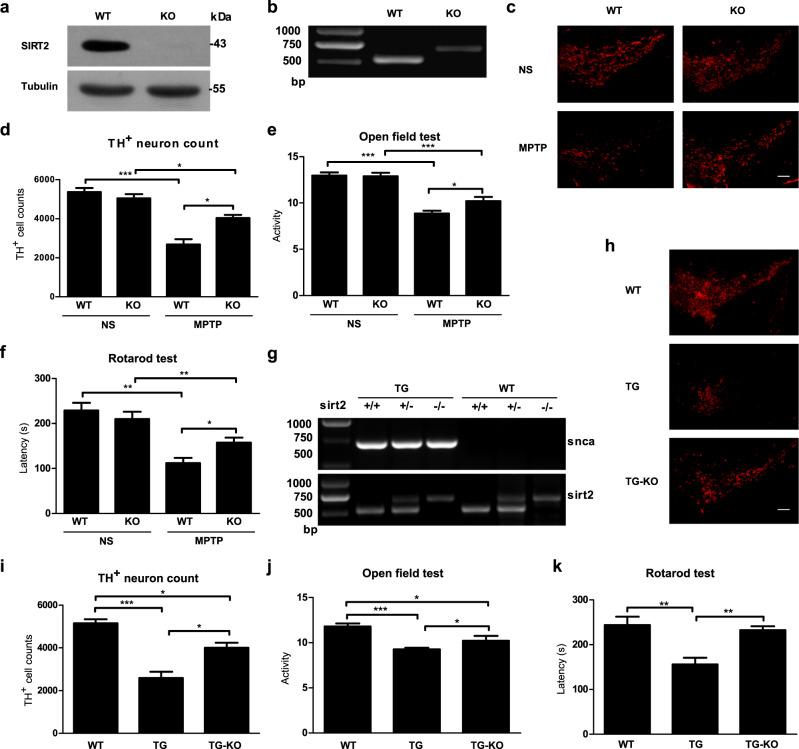
SIRT2 deletion protects against α-synuclein-A30P*A53T transgenic and MPTP-induced mouse model of PD. **a** Western blotting was performed to examine the protein expression of SIRT2 in wild-type (WT) and SIRT2 knockout (KO) mice. **b** Reverse-transcription PCR (RT-PCR) was used to detect the mRNA expression of Sirt2 in WT and SIRT2 KO mice. **c** Immunostaining was performed to detect tyrosine hydroxylase (TH)-positive neurons in the substantia nigra pars compacta (SNpc) in MPTP-treated WT and SIRT2 KO mice (the control group was treated with normal saline (NS)). The scale bar represents 200 μm. **d** The number of TH-positive neurons in the SNpc was counted (*n* = 3). **e** The open field test and **f** Rotarod test were used to examine MPTP-treated WT and SIRT2 KO mice (*n* = 15). **g** RT-PCR was employed to assess the mRNA expression of α-synuclein and Sirt2 in WT mice, α-synuclein-A30P*A53T transgenic (TG) mice, and α-synuclein-A30P*A53T transgenic mice with SIRT2 knockout (TG-KO). **h** Immunostaining was performed to detect TH-positive neurons in the SNpc in WT, TG, and TG-KO mice. The scale bar represents 200 μm. **i** Numbers of TH-neurons in the SNpc were counted stereologically (*n* = 3). **j** The open field test and **k** rotarod test were used to detect motor deficits in WT, TG, and TG-KO mice (*n* = 8). All data are presented as the means ± SD. Statistical analyses were conducted using two-way ANOVA followed by Tukey’s post hoc test in (**d**–**f**). Statistical analyses were conducted using one-way ANOVA followed by Tukey’s post hoc test in (**i**–**k**). *P < 0.05, **P < 0.01, ***P < 0.001.

To determine whether the ablation of SIRT2 can rescue PD effects, TH-positive neurons within the SNpc were examined in SIRT2 KO (knockout) mice after the systemic administration of 1-methyl-4-phenyl-1,2,5,6-tetrahydropyrine (MPTP, i.p.). Further, behavioral tests were also performed. The protein and mRNA expression of SIRT2 was verified in wild-type (WT) and KO mice (Fig. 1 a, b). Indeed, there was no significant difference in behavioral outcomes between the WT and KO groups (Fig. S1a–i). However, SIRT2 KO mice treated with MPTP (i.p.) showed a higher number of DA neurons within the SNpc (Fig. 1c, d) than did WT mice treated with MPTP. They also showed better performance on PD-related behavioral tests, such as the open field test (Fig. 1e) and rotarod test (Fig. 1f). Furthermore, a α-synuclein-A30P*A53T double mutant transgenic model of PD lacking SIRT2 was also generated (Fig. 1g). As observed in the MPTP-induced PD model, 18-month-old α-synuclein-A30P*A53T transgenic mice with SIRT2 knockout exhibited an attenuated decrease in DA neurons in the SNpc (Fig. 1h, i). Further, they also showed significantly ameliorated behavioral phenotypes (Fig. 1j, k). Together, these results indicated that SIRT2 KO in PD mouse models leads to a significant improvement in behavioral phenotypes and reduction in DA neuron loss.

### SIRT2 expression is not altered in PD models

**Fig. 2:**
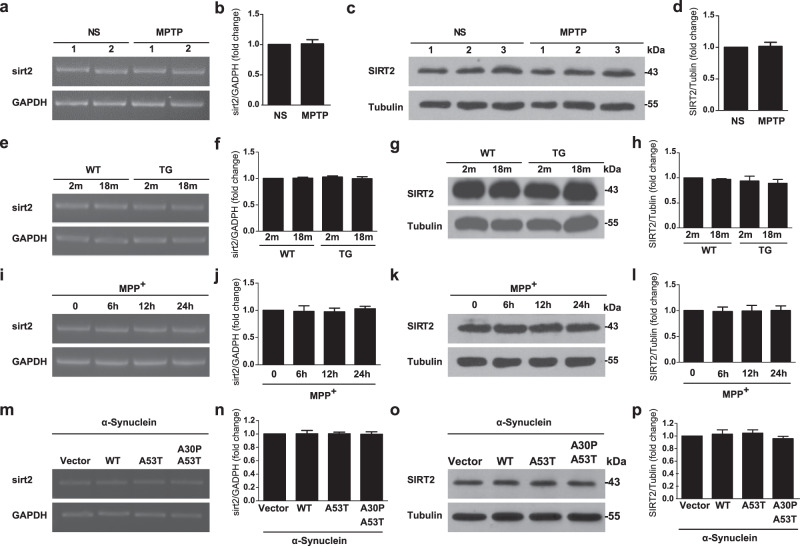
SIRT2 expression remains unchanged in PD models. **a** The mRNA levels of SIRT2 in the SNpc were examined using RT-PCR in mice treated with normal saline (NS) or MPTP. **b** Quantification of SIRT2 mRNA levels shown in (**a**) (*n* = 3). **c** The protein levels of SIRT2 in the SNpc were examined using western blotting in mice treated with NS or MPTP. **d** Quantification of SIRT2 protein levels shown in (**c**) (*n* = 3). **e**–**h** mRNA and protein levels of SIRT2 in the SNpc assessed were assessed using RT-PCR and western blotting (*n* = 3), respectively, in WT and α-synuclein-A30P*A53T transgenic (TG) mice. **i**–**l** mRNA and protein levels of SIRT2 were examined in primary culture neurons treated with 50 μM MPP^+^ for 0, 6, 12, and 24 h using RT-PCR and western blotting (*n* = 3). **m**–**p** SIRT2 expression was examined in SY5Y cells stably expressing GFP vector, GFP-α-synuclein, GFP-α-synuclein-A53T, and GFP-α-synuclein-A30P*A53T (*n* = 3). All data are presented as the means ± SD. Statistical analyses were conducted using an unpaired *t*-test in (**b**) and (**d**). Statistical analyses were conducted using two-way ANOVA followed by Tukey’s post hoc test in (**f**) and (**h**). Statistical analyses were conducted using one-way ANOVA followed by Tukey’s post hoc test in (**j**, **l**, **n**, **p**). None of the differences were significant.

Given the protective role of SIRT2 KO in transgenic mouse models of PD, we studied whether SIRT2 expression becomes altered during PD progression. First, using reverse-transcription polymerase chain reaction (RT-PCR) and immunoblotting, we confirmed that the mRNA and protein levels of SIRT2 were not altered in the SNpc in the MPTP-induced (WT mice) (Fig. 2a–d) and α-synuclein-A30P*A53T overexpression (transgenic mice) models of PD (Fig. 2e–h). Additionally, SIRT2 mRNA and protein levels were also unchanged in two cellular models of PD, i.e., MPP^+^ (1-methyl-4-phenylpyridinium)-treated primary culture neurons (Fig. 2i–l) and SH-SY5Y cells stably transfected with α-synuclein-A30P*A53T (Fig. 2m–p). Harrison et al.^14^ demonstrated that SIRT2 expression in the SNpc of PD brains remains relatively unchanged between either early or late PD cases compared from controls. The findings from our PD model were consistent with their findings. Thus, these data strongly suggest that SIRT2 expression is not altered during the pathogenesis of PD.

### Nuclear–cytoplasmic shuttling of SIRT2

**Fig. 3:**
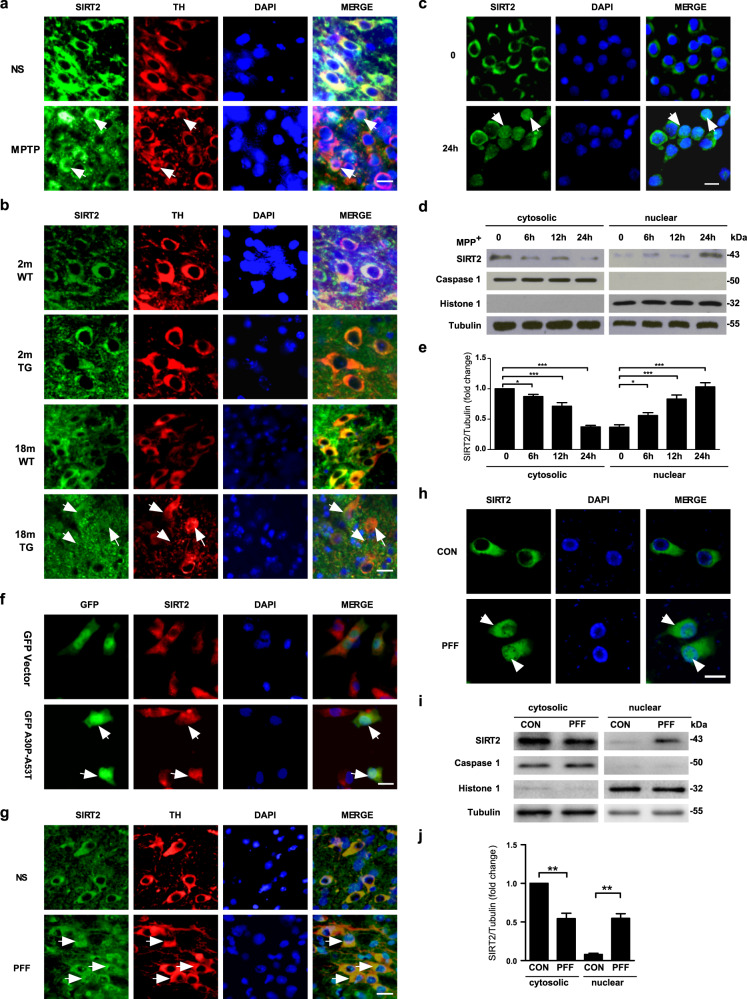
SIRT2 translocates from the cytoplasm to the nucleus in PD. **a**, **b** Subcellular localization of SIRT2 was monitored using dual immunolabeling for TH (red) and SIRT2 (green) in the SNpc in mice treated with normal saline (NS) or MPTP (**a**) as well as in WT and α-synuclein-A30P*A53T transgenic mice (TG) (**b**). **c** Immunofluorescence assays for SIRT2 (green) were conducted in primary culture neurons treated with 50 μM MPP^+^ for 24 h. **d** The localization of SIRT2 was detected using nuclear/cytosolic immunoblotting in primary culture neurons treated with 50 μM MPP^+^ for 0, 6, 12, and 24 h. **e** Quantification of SIRT2 protein levels in the cytoplasm and nucleus (separate from **d**). **f** SH-SY5Y cells were transiently transfected with GFP vector (green) or GFP-α-synuclein-A30P*A53T (green) plasmids for 24 h and subsequently immunostained for SIRT2 (red) and observed using confocal microscopy. **g** Subcellular localization of SIRT2 examined using dual immunolabeling for TH (red) and SIRT2 (green) in the mouse SNpc 35 days after the striatal stereotactic injection of 2 μl (per side) normal saline (NS), or α-synuclein PFF (Abcam, ab246002, 1 μg/μl). **h** Localization of SIRT2 detected using nuclear/cytoplasmic immunofluorescence staining in primary culture neurons treated with 4 μg/ml PFF for 7 days. **i** Localization of SIRT2 detected using nuclear/cytosolic immunoblotting in primary culture neurons treated with 4 μg/ml PFF for 7 days. **j** Quantification of SIRT2 protein levels in the cytoplasm and nucleus (separate from [**i**]). The scale bar represents 20 μm. All data are presented as the means ± SD. Statistical analyses were conducted using one-way ANOVA followed by Tukey’s post hoc test in (**e**). Statistical analyses were conducted using two-way ANOVA followed by Tukey’s post hoc test in (**j**). *P < 0.05, **P < 0.01, ***P < 0.001.

The findings of our study showed that SIRT2 deletion provides protection against PD. However, the protective mechanism was unclear. SIRT2 is predominantly expressed in the cytosol; however, SIRT2 translocation to the nucleus has been observed during bacterial infection^15^ and cell mitosis^16^. To explore the subcellular localization of SIRT2 in PD, we evaluated the distribution of SIRT2 in TH-positive neurons from the SNpc in mouse models of PD using double immunofluorescence staining. Surprisingly, we found that nuclear SIRT2 levels were increased in the MPTP-induced mouse model of PD (Fig. 3a and Fig.S1j–k) and in 18-month-old α-synuclein-A30P*A53T transgenic mice (Fig. 3b). Consistent with these findings, we also observed that SIRT2 translocated from the cytosol to the nucleus in primary culture neurons treated with MPP^+^ (Fig. 3c–e and Fig. S1l). Similar results indicative of SIRT2 shuttling were also observed in SH-SY5Y cells transiently transfected with α-synuclein-A30P*A53T (Fig. 3f and Fig. S1m). To ensure that SIRT2 translocation from the cytoplasm to the nucleus is conserved in PD, these findings were replicated in a pre-clinical model of PD (PFF model, Fig. S2a-g and Fig. 3g–j). We found that the nuclear localization of SIRT2 increased in PFF-treated mice (Fig. 3g) and primary culture neurons (Fig. 3h–j and Fig. S2g). SIRT2 translocation was also elevated in primary midbrain DA neurons treated with MPP^+^ or PFF (Fig. S3a). Collectively, the results demonstrated that SIRT2 translocates from the cytoplasm to the nucleus in PD.

### Nuclear localization of SIRT2 promotes neuronal death

**Fig. 4:**
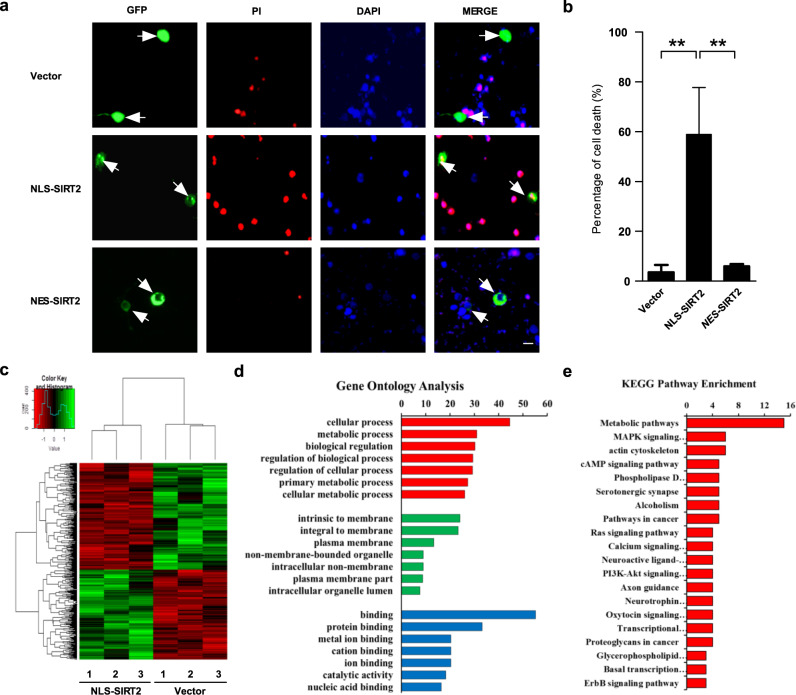
Nuclear localization of SIRT2 promotes neuronal death. **a** Primary culture neurons were transiently transfected with GFP vector or GFP-NLS-SIRT2 plasmids and subsequently stained using anti-SIRT2 antibodies (green), propidium iodide (PI) (red), and DAPI (blue). The scale bar represents 10 μm. **b** GFP-positive cells (green) that also showed positive PI staining (red) were counted as dead neurons. The percentage of PI-positive cells (red) among at least 100 GFP-positive cells was measured for each group (*n* = 3). All data are presented as the means ± SD. Statistical analyses were conducted using one-way ANOVA followed by Tukey’s post hoc test, and ** represents *P* < 0.01. **c** HEK293 cells were transfected with GFP vector and GFP-NLS-SIRT2 plasmids for 24 h and subsequently collected for RNA-seq. The differentially expressed genes between the two groups are illustrated using a heatmap (*n* = 3). The colors indicate relative expression levels (red, high expression; green, low expression). **d** Gene Ontology (GO) terms. Frequency of sequences with assigned GO terms for the biological process category across the different samples. **e** KEGG pathway enrichment. Biological pathways associated with the differentially expressed genes in the different samples.

SIRT2 was found to translocate from the cytosol to the nucleus in PD. However, the effect of this nuclear localization was still unknown. To further explore the effects of nuclear SIRT2 localization, we transfected primary culture neurons with a GFP-tagged SIRT2 plasmid containing an NLS (termed nuclear location sequence). A single cell death count assay indicated that NLS-SIRT2 transfection led to higher cell mortality than did transfection of the GFP vector, indicating that nuclear SIRT2 promoted neuronal death (Fig. 4a, b). Meanwhile, cytoplasmic SIRT2 (NES-SIRT2) had no influence on cell survival in primary cortical neurons (Fig. 4a, b). Next, we sought to obtain further evidence on the targets of nuclear SIRT2 and the genes/pathways it regulates. HEK293 cells, which are widely used in molecular and cellular neurobiology, were used for RNA sequencing (RNA-seq) after the overexpression of GFP-tagged NLS-SIRT2. The transfection efficiency in HEK293 cells was more than 80%. Overall, RNA-seq data showed that a total of 424 genes (194 up- and 230 down-regulated genes) were differentially expressed between cells transfected with the NLS-SIRT2 and vector constructs (Fig. 4c). These 424 differentially expressed genes (DEGs) were subjected to Gene Ontology (GO) enrichment analysis based on the number of unigene alignments (Fig. 4d). In GO analysis, three major functional categories were assessed: biological process, cellular component, and molecular function. The DEGs were mainly related to cellular processes and intrinsic membrane and protein binding. Kyoto Encyclopedia of Genes and Genomes (KEGG) pathway analysis revealed the top 20 enriched pathways, with metabolic pathway, MAPK signaling pathway, and actin cytoskeleton showing the three highest enrichment values (Fig. 4e). To verify the reliability of the RNA-seq analysis, the target genes of the MAPK signaling pathway—MAPK7 and PLA2G4A—were selected as representative genes. The expression of these genes was verified using qPCR in neuronal PD models (MPP^+^, PFF). qPCR showed that the expression level of MAPK7 in the neuronal PD model group was higher than that in the control group (Fig. S3b, c), whereas the level of PLA2G4A was lower (Fig. S3d, e). These qPCR results for MAPK7 and PLA2G4A expression in the neuronal PD model were consistent with those of the RNA-seq analysis.

### Cdk5 is involved in SIRT2 nuclear translocation

**Fig. 5:**
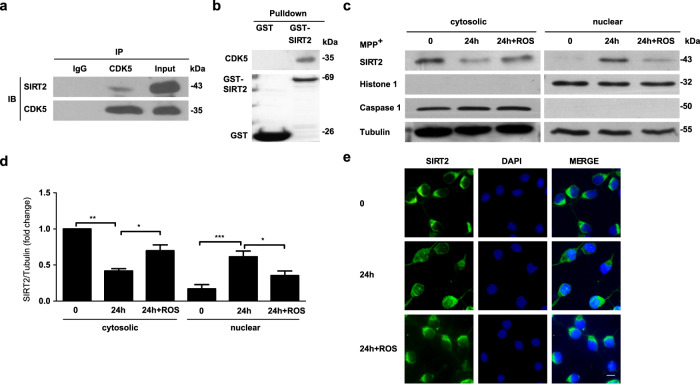
Cdk5 is involved in the nuclear translocation of SIRT2. **a** Co-Immunoprecipitation (Co-IP) was performed to identify whether endogenous SIRT2 interacts with Cdk5. **b** A GST-pull-down assay was used to verify whether exogenous SIRT2 can bind to Cdk5. **c** Cultured primary neurons were pretreated with 10 μM roscovitine (ROS) for 0.5 h and subsequently treated with 50 μM MPP^+^ for 24 h. The cytoplasmic and nuclear distribution of SIRT2 was detected using immunoblotting. **d** Quantification of SIRT2 protein levels in the cytoplasm and nucleus shown in **c** (*n* = 3). **e** Immunofluorescence staining for SIRT2 (green) was performed to observe the distribution of SIRT2 in primary culture neurons subjected to ROS stress. The scale bar represents 10 μm. All data are presented as the means ± SD. Statistical analyses were conducted using one-way ANOVA followed by Tukey’s post hoc test in **d**. *P < 0.05, **P < 0.01, ***P < 0.001.

Cdk5 has been implicated in the regulation of multiple cellular events involved in PD pathogenesis^17^. Furthermore, Cdk5 activation has been observed in MPTP-treated mice and transgenic models of PD in numerous studies by other researchers^18,19^ and our research group^20,21^. In an effort to clarify the precise molecular regulation of the SIRT2 protein in PD, we examined whether the critical serine/threonine kinase Cdk5 is involved in SIRT2 nuclear translocation. GST pull down and co-immunoprecipitation assays were conducted, and it was found that Cdk5 can interact with endogenous (Fig. 5a) and exogenous SIRT2 (Fig. 5b). Furthermore, primary culture neurons were treated with the known Cdk5 kinase inhibitor, roscovitine (Ros). Immunoblotting (Fig. 5c, d) and immunofluorescence (Fig. 5e and Fig. S3f) showed that such treatment markedly suppressed the nuclear translocation of SIRT2. Moreover, Ros also significantly inhibited the nuclear translocation of SIRT2 in primary midbrain DA neurons treated with MPP^+^ or PFF (Fig. S3a). Furthermore, it rescued the neuronal death induced by MPP^+^ (Fig. S3g).

### Phosphorylation of SIRT2 by Cdk5 mediates its nuclear translocation

**Fig. 6:**
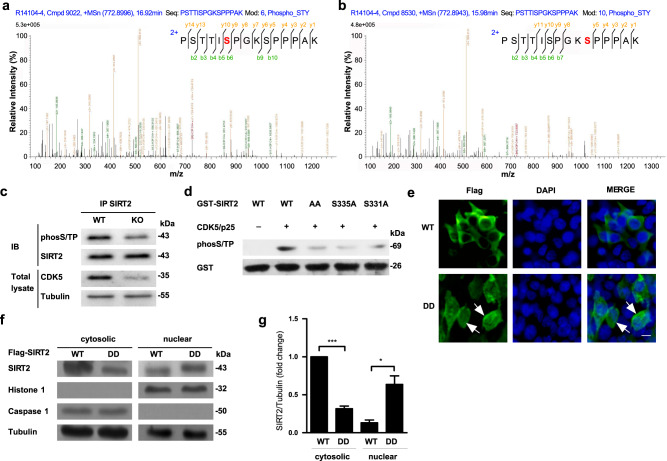
Cdk5 phosphorylates SIRT2 and thereby mediates the nuclear translocation of SIRT2. **a** Liquid chromatography-mass spectrometry after a kinase assay in vitro demonstrated that the Ser331 and **b** Ser335 sites of SIRT2 were phosphorylated by Cdk5. **c** Phosphorylation levels of SIRT2 were detected in forebrain cortical tissue obtained from forebrain neuron-specific Cdk5 knockout mice and WT mice. Phosphorylation of SIRT2 was detected in primary culture neurons using immunoprecipation and western blotting with anti-phosS/TP and anti-SIRT2 antibodies. **d** SDS-PAGE used for the Cdk5/p35 kinase assay in vitro. Purified GST-SIRT2 WT, S331A, S335A, and S331AS335A (AA) fusion proteins were mixed with active Cdk5/p35 and ATP, and the reaction mixture was analyzed using western blotting with anti-phosS/TP and anti-GST antibodies. **e** HEK293 cells were transiently transfected with Flag-SIRT2 WT or Flag-SIRT2 mutation DD (double mutations at S331D and S335D) plasmids for 24 h, and subsequently, the cytoplasmic and nuclear distribution of SIRT2 (green) was detected using immunofluorescence staining. The scale bar represents 10 μm. **f** Immunoblotting assays for SIRT2 was performed using an anti-Flag antibody in HEK293 cells transfected with Flag-SIRT2 WT or Flag-SIRT2-DD. **g** Quantification of SIRT2 protein levels shown in **f** (*n* = 3). All data are presented as the means ± SD. Statistical analyses were conducted using two-way ANOVA followed by Tukey’s post hoc test in **g**. *P < 0.05, ***P < 0.001.

To demonstrate that SIRT2 was a Cdk5 substrate, we performed prediction analysis with GPS5.0 (ref. ^22^; Fig. S4a) and Scansite 3beta^23^ (Fig. S4b). Two serine residues (Ser331 and Ser335) in SIRT2 were predicted to be phosphorylation sites. Meanwhile, multiple alignment analysis also indicated that the protein sequence of SIRT2 was extremely conserved in disparate species (Fig. S4c). Using liquid chromatography-mass spectrometry, we confirmed that Ser331 and Ser335 could both be phosphorylated by Cdk5 in vitro (Fig. 6a, b). Consistent with these in vitro findings, we also confirmed that Cdk5 is required for SIRT2 phosphorylation in vivo. The phosphorylation levels of Cdk5 and SIRT2 in the SNpc were significantly higher in mice treated with MPTP or PFF than in controls (Fig. S5a–h). The phosphorylation levels of SIRT2 showed a significant reduction in forebrain cortical tissue after the neuron-specific conditional knockout of Cdk5 (Fig. 6c). In addition, an in vitro Cdk5 kinase assay was conducted using a GST-tagged purified fusion SIRT2 protein. While GST-SIRT2 was robustly phosphorylated, GST-SIRT2-AA (double mutant with phosphor-nonactive S331A and S335A) was not (Fig. 6d).

To assess whether SIRT2 phosphorylation by Cdk5 regulates its nuclear translocation, we constructed a SIRT2 protein with a phosphomimic (continuously active state) mutation (SIRT2-DD, double mutation at S331D and S335D). As expected, immunofluorescence staining showed that overexpressed SIRT2-DD exhibited notably higher nuclear translocation than SIRT2-WT in HEK293 cells lacking the molecular chaperone of Cdk5 (Fig. 6e). Moreover, using immunoblotting assays, a similar result was observed in cells transfected with SIRT2-DD (Fig. 6f, g). To further explore the role of SIRT2-DD, we transfected primary culture neurons with the SIRT2-DD plasmid. A single-cell death counting assay indicated that SIRT2-DD increased cell mortality, resulting in higher neuronal death than the empty vector and SIRT2-WT plasmid (Fig. S6a, b). Taken together, these data suggested that Cdk5 is an important regulator of SIRT2 nuclear translocation and acts by directly phosphorylating the Ser331 and Ser335 residues of SIRT2.

### Myr-SIRT2_328–339_ interference peptide rescues neuronal loss and motor dysfunction

**Fig. 7:**
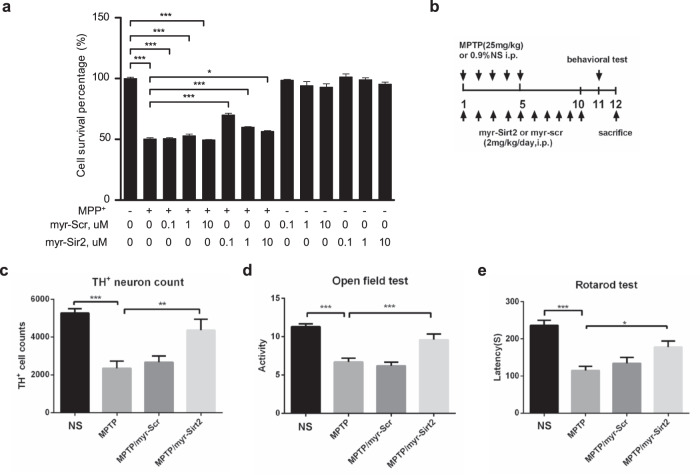
Myr-SIRT2_328–339_ interference peptide rescues neuronal death and motor behavior. **a** Primary culture neurons were pretreated with the SIRT2_328–339_ or Scramble peptide conjugated with Myristic acid (Myr) and subsequently treated with 100 μM MPP^+^ for 48 h. The survival percentage of neurons was detected using an MTT assay. **b**, **c** Immunostaining for TH in the SNpc, **d** the open field test, and **e** the rotarod test were performed in MPTP-treated mice who received Myr-SIRT2_328–339_ at a dose of 2 mg/kg (i.p.) once per day. The scramble peptide was used as the control. The number of TH-positive neurons in the SNpc was counted (Myr-SIRT2_328–339_, *n* = 3; Scramble, *n* = 4). The behavioral tests were conducted in 10 (Myr-SIRT2_328–339_) and 12 mice (Scramble) each. All data are presented as the means ± SD. Statistical analyses were conducted using one-way ANOVA followed by Tukey’s post hoc test in (**a**, **c**–**e**). *P < 0.05, **P < 0.01, ***P < 0.001.

Based on our findings, we designed a short peptide named Myr-SIRT2_328–339_, which competitively inhibits Cdk5-dependent Ser331 and Ser335 phosphorylation in SIRT2. This peptide was conjugated to myristic acid to ensure its cell-penetrating ability^24^. Unsurprisingly, Myr-SIRT2_328–339_ decreased the mortality of MPP^+^-treated primary culture neurons (Fig. 7a). Furthermore, immunofluorescence showed that the treatment of primary culture neurons with Myr-SIRT2_328–339_ markedly suppressed the MPP^+^-induced nuclear translocation of SIRT2 (Fig. S7a). Compared to treatment with MPP^+^ alone, treatment with MPP^+^ combined with SIRT2_328–339_ peptide pretreatment resulted in lower SIRT2 phosphorylation levels in primary culture neurons (Fig. S7b, c). Given the protective efficacy of the Myr-SIRT2_328–339_ peptide in vitro, we assessed its effect on SNpc DA neuron loss and motor function performance in MPTP-treated WT mice. Consistent with the findings in MPP^+^-treated primary cortical neurons, the Myr-SIRT2_328–339_ peptide group exhibited reduced DA neuron loss (Fig. 7b, c), increased locomotory activity (Fig. 7d), and improved motor function (Fig. 7e). Overall, the results showed that Cdk5-dependent phosphorylation of SIRT2 mediates its nuclear translocation, and competitive inhibition of SIRT2 phosphorylation can attenuate neuronal death in an MPP^+^-treated cellular model and MPTP-treated mouse model of PD.

## Discussion

The dysregulation of several Sirtuin proteins is linked to a series of neurological disorders, including PD. SIRT1 and SIRT2, primarily expressed in the nucleus and cytoplasm, respectively, are believed to have a “yin-yang” relationship in neurodegeneration^25^. Although SIRT1 is predominantly expressed in the nucleus, the nuclear and cytoplasmic shuttling of SIRT1 has been demonstrated to affect different deacetylation substrate proteins. Thus, strong evidence suggests that SIRT1 has different but specific functions depending on its subcellular localization^26^. Notably, the primarily cytoplasmic localization of SIRT2 is similar to SIRT1 that displays nuclear import in many biological processes. Consistent with the protective role of SIRT1 activation, our study provides powerful evidence that SIRT2 deletion using gene knockout can effectively alleviate DA neuron death and PD-related motor symptoms, both in an environmental (MPTP drug-induced model) and genetic model (α-synuclein-A30P*A53T transgenic model). Meanwhile, several nuclear substrates deacetylated by SIRT2 have been implicated in diseases, including p53, FOXO1, FOXO3a, histone H4, histone H3, and p300 (ref. ^27^). More research is needed to better understand the detailed relationship between the deacetylation of these substrates and the nuclear localization of SIRT2.

Cdk5, a multifaceted kinase, has been linked to an array of disorders in neuronal migration, neuronal differentiation, synapse development, and synaptic function and even to neuronal death^17^. Evidence suggests that Cdk5-mediated phosphorylation of diverse substrates is crucial for controlling neuronal cell cycle re-entry during progressive neuronal death in PD^17,28^. In this study, we demonstrated one molecular mechanism of Cdk5 action. We showed that Cdk5-dependent SIRT2 phosphorylation mediates the nuclear translocation of SIRT2. This mechanism appears to be key for cell cycle re-entry and neuronal death and can be targeted to enhance protection against DA neuronal loss in PD. As expected, the myristic acid-conjugated short peptide Myr-SIRT2_328–339_ could specifically inhibit Cdk5-mediated phosphorylation at the Ser331 and Ser335 sites of SIRT2. This inhibition significantly attenuated the loss of primary culture neurons treated with MPP^+^, a classical PD model inducer in cells. Additionally, the translocation of SIRT2 from the cytoplasm to the nucleus was dependent on classic nuclear import pathways and required binding with importin-α and importin-β. Interestingly, the results showed that Cdk5-dependent phosphorylation at Ser331 and Ser335 is essential for the nuclear import of SIRT2. The findings obtained in this study mirror those of previous studies examining the pivotal role of protein phosphorylation in the regulation of nucleo-cytoplasmic trafficking^29^.

Although a number of studies, including the present one, have indicated that SIRT2 deletion or inhibition could promote neuroprotection in PD^14,30,31^, the precise mechanism through which SIRT2 mediates the death of DA neurons has yet to be elucidated. Interestingly, our results suggest that SIRT2 translocates from the cytoplasm to the nucleus, and the nuclear localization of SIRT2 promotes neuronal death. However, cytoplasmic SIRT2 produces no neurotoxicity. Indeed, one study has suggested that the nuclear translocation of SIRT2 causes a marked decrease in H4K16 deacetylase levels, leading to cell cycle re-entry and subsequent neuronal death^32^. Importantly, another previous study has shown that SIRT2 plays a key role in cell cycle regulation^33^. These studies are consistent with our results and suggest that the nuclear localization of SIRT2 is a crucial regulator of mitotic cells during mitosis owing to the deacetylase activity of SIRT2. Nevertheless, central nervous system neurons are typically considered postmitotic cells, which was probably nothing permanent about the neuronal cell cycle arrest. Additionally, the role of cell cycle re-entry in neurons is generally considered as a common apoptotic mechanism of neuronal cell death^34^. Our results showed that SIRT2 translocates from the cytosol to the nucleus and contributes to cell cycle re-entry in DA neurons, leading to neuronal death in PD. Furthermore, our RNA-seq-based KEGG pathway enrichment analysis indicated that SIRT2 mainly regulates metabolic pathways, the MAPK signaling pathway, and the actin cytoskeleton. Growing evidence indicates that PD is a disease of metabolic dysfunction, and controlling specific pathways such as those involved in glutathione metabolism^35^, nucleotide metabolism^36^, and glucose metabolism^37^ could protect against neuronal loss in PD. The MAPK signaling pathway, which showed the second highest enrichment in our KEGG pathway analysis, is involved in regulating a diverse array of cellular functions, including cell proliferation, differentiation, and mitosis^38^. We found that the target genes of the MAPK signaling pathway, such as FGF12, RRAS2, PLA2G4A, MAPK7, SOS2, and CACNA1B, were regulated by nuclear SIRT2. These genes may be involved in controlling the cell cycle at the G2 to M checkpoint, leading to cellular proliferation in mitotic cells but cell cycle re-entry in postmitotic neurons. Furthermore, our results—which show that SIRT2 mainly regulates cell cycle re-entry—could explain why SIRT2 inhibition has contrasting effects in *Drosophila* models^30^ and MPP^+^-induced PC12 cellular models of PD^39^. In postmitotic neurons, nuclear SIRT2 could promote cell cycle re-entry and subsequently cause neuronal death. Conversely, in mitotic cells, such as PC12 cells, nuclear SIRT2 would predominantly promote cell mitosis and proliferation by regulating the cell cycle. Consistent with the present results, our previous study also demonstrated that neuronal cell cycle re-entry contributes to the biological process of neuronal death in PD^20^. Thus, our results uncover a novel mechanism of neuronal death regulation via the nuclear–cytoplasmic shuttling of SIRT2 during PD progression, Nevertheless, the detailed mechanism underlying the effect of nuclear SIRT2 on neuronal death warrants further research.

In conclusion, the present study is the first to demonstrate the important neurotoxic effects of nuclear–cytoplasmic SIRT2 shuttling, which is dependent on Cdk5-mediated phosphorylation, during neuronal death in PD. Future studies aimed at clinically validating the efficacy and safety of the polypeptide drug Myr-SIRT2_328–339_ are essential for demonstrating that SIRT2 inhibition can prevent DA neuronal death in patients with PD. Nevertheless, our study provides strong evidence suggesting the potential of SIRT2 as a therapeutic target in PD.

## Methods

### Antibodies and chemical reagents

The following primary antibodies were used. The rabbit polyclonal anti-Sirt2 antibody (s8447, WB-1:4000, IF-1:200) was purchased from Sigma-Aldrich. The rabbit polyclonal anti-Caspase-1 (ab74279, WB-1:1000) and anti-phosphothreonine-proline/phosphoserine-proline (ab9344, WB-1:5000) antibodies were purchased from Abcam. The mouse monoclonal anti-Histone-1 (sc393358, WB-1:1000), mouse monoclonal anti-TH (sc-374047, WB-1:1000), and mouse monoclonal anti-Cdk5 (sc-6247, WB-1:1000) antibodies were purchased from Santa Cruz. The mouse monoclonal anti-glutathione *S*-transferase (GST) (CST2624, IF-1:100) and mouse monoclonal anti-β-tubulin (KM9003, WB-1:4000) antibodies were obtained from Tianjin Sungene Biotech.

The following secondary antibodies were used. The Peroxidase AffiniPure Goat anti-mouse IgG (WB-1:20000), DyLight 594-conjugated AffiniPure goat anti-mouse IgG (IF-1:400), Peroxidase AffiniPure goat anti-rabbit IgG (WB-1:20000), and DyLight 488-conjugated AffiniPure goat anti-rabbit lgG (IF-1:400) antibodies were obtained from Jackson ImmunoResearch. 1-Methyl-4-phenylpyridiniumiodide (MPP+), 1-methyl-4-phenyl-1,2,3,6-tetrahydropyridine hydrochloride (MPTP), cytosine arabinoside (AraC), propidium iodide (PI) and 4′,6-diamidino-2-phenylindole dihydrochloride (DAPI) were obtained from Sigma-Aldrich. DAPI and PI were used for nuclear counterstaining at a concentration of 1 mg/ml and 10 μg/ml, respectively. Recombinant mouse alpha-synuclein protein preformed fibril (Active) (PFF, ab246002) was obtained from Abcam.

### Animals

Sirt2 knockout (Sirt2 KO, stock number: 012772) mice (background: C57BL/6J) were used in the study. Exons 5–6 and a part of exon 7 in the Sirt2 gene were replaced with a floxed neomycin (neo)-resistance cassette. C57BL/6J-Tg (Th-SNCA*A30P*A53T) 39Eric/J mice (Tg-SNCA, stock number: 008239) were also used in the present study as a transgenic mouse model of PD. Brain-specific Cdk5 conditional knockout (cKO) mice were generated by crossing B6.129S4 (Cg)-Cdk5tm1.1Lht/J mice (Cdk5-loxP, stock number: 014156) with CaMKII-iCre mice (J012362, purchased from the Model Animal Research Center of Nanjing University). Sirt2 KO, Cdk5 KO, and Tg-SNCA mice were purchased from Jackson Laboratories. All mice were genotyped using DNA extracted from tail tips and subsequent PCR assays based on the genotyping protocol database of The Jackson Laboratory website. Sirt2 KO mice were genotyped using all three of the following primers: sirt2-10475, 5′-GAC TGG AAG TGA TCA AAG CTC-3′; sirt2-10476, 5′-CAG GGT CTC ACG AGT CTC ATG-3′; and sirt2-10477, 5′-TCA AAT CTG GCC AGA ACT TAT G-3′ (mutant = +:700 bp, heterozygote = 538 bp and 700 bp, and WT = 538 bp). Tg-SNCA mice were genotyped using two primers: Tg-SNCA-F, 5′-CAG GTA CCG ACA GTT GTG TAA AGG AAT-3′ and Tg-SNCA-R, 5′-GAT AGC TAT AAG GCT TCA GGT TCG TAG TCT-3′ (transgene=469 bp, WT = no bands). Cdk5 KO mice were genotyped using two primers: Cdk5-loxP-F, 5′-CAG TTT CTA GCA CCC AAC TGA TGT A-3′ and Cdk5-loxP-R, 5′-GCT GTC CTG GAA CTC CAT CTA TAG A-3′ (mutant = 460 bp, heterozygote = 460 and 660 bp, and WT = 660 bp). Cycling conditions were as follows: 97 °C for 3 min (97 °C for 30 s, 65 °C for 30 s, and 72 °C for 30 s) × 35, and 2 min at 72 °C. Animals were housed under standard conditions with a 12-h light/12-h dark cycle and unlimited access to water and chow. Mice were treated with 25 mg/kg MPTP or an equivalent volume of 0.9% saline via intraperitoneal injection once a day for five consecutive days, as previously reported^20,21^. Substantia nigra and forebrain tissue were extracted for RT-PCR and western blotting. All animal experiments were performed according to protocols approved by the Institutional Animal Ethics Committee.

### Stereotactic injection

Eight-week-old mice were deeply anesthetized using 5% isoflurane, and anesthesia was maintained at 0.25–4% during surgery. Then, 2 μl of the recombinant mouse alpha-synuclein protein PFF (Abcam, ab246002) was injected unilaterally into the dorsolateral striatum (AP: 0.2 mm from the bregma; ML: −2 mm from the midline; DV: −2.6 mm below the dura) using a glass microelectrode (flow rate, 0.250 μl/min). After surgery, the mice were allowed to recover on a heating pad for at least 15 min. All mice were supervised until they were fully awake. Mice were sacrificed 35 days after injection, and tissue samples were obtained.

### Plasmids and transfection

The Flag-SIRT2 (pcDNA3.1^+^), GST-SIRT2 (pGEX-4T-3), GFP vector (pEGFP-C1), and GFP-NLS-Sirt2 plasmids were a kind gift from Prof. Xue-min Wang (Southern Medical University). The GFP-α-synuclein, GFP-α-synuclein-A53T, GFP-α-synuclein-A30P*A53T, GST-SIRT2-S331A, GST-SIRT2-S335A, GST-SIRT2-S331AS335A, and Flag-SIRT2-S331DS335D constructs were synthesized in our laboratory.

### Primary cortical neuron culture

Dissociated primary cortical neurons were isolated from rat embryos at embryonic day 16–18. They were cultured on six-well plates coated with poly-l-lysine (0.1 mg/ml) in Neurobasal medium supplemented with 2% B27 (Invitrogen). After 24 h, the cell division inhibitor AraC was added to the culture media at a final concentration of 10 μM. All treatments were performed 7 days after plating.

### Culture of cell lines

SY5Y cells stably overexpressing GFP vector, GFP-α-synuclein, GFP-α-synuclein-A53T, and GFP-α-synuclein-A30P*A53T were produced in our laboratory. Subsequently, the somatic localization of SIRT2 was detected using RT-PCR and immunoblotting. For RT-PCR, SH-SY5Y cells were transiently transfected with the GFP vector or GFP-α-synuclein-A30P*A53T plasmid. After 24 h, RT-PCR was performed, and subsequently, the somatic localization of SIRT2 was detected by immunolabeling. In order to identify the subcellular localization of SIRT2 using RT-PCR and western blotting, SH-SY5Y cells stably transfected with GFP-α-synuclein-A30P*A53T were generated from GFP-positive specific single-cell-derived clones using G418. HEK293 cells were transfected with the Flag-SIRT2 WT or Flag-SIRT2 mutation DD (double mutations at S331D and S335D) plasmid. After 24 h, the somatic localization of SIRT2 was detected using nucleus/cytoplasm immunoblotting and immunolabeling.

### Single cell death assay

Seven days after initial plating, primary cortical neurons were transfected with a GFP vector plasmid or a GFP-NLS-SIRT2 using Lipofectamine 2000 (Invitrogen) according to the manufacturer’s instructions. PI was added 1 h before the end of the transfection duration. After 24 h of transfection, the proportion of dead primary neurons was calculated based on the number of cells showing PI (red) and GFP co-localization as a function of the total number of GFP-positive cells (green), as described previously^40^.

### RT-PCR

Total RNA was isolated from MPP^+^-treated neurons and substantia nigra tissue obtained from mice using the TRIzol reagent (Takara). Then, 1 μg of total RNA from each sample was reverse transcribed using the TransScript All-in-One First-Strand complementary DNA (cDNA) Synthesis SuperMix for PCR (TransGene Biotech) (total volume, 20 μl). For PCR, amplification was carried out in a total reaction volume of 20 μl (1 μl of each primer, 10 μl of 2× Taq Master Mix, 1 μl of cDNA, and 7 μl of RNA-free water) according to the manufacturer’s instructions. The sequences of forward (F) and reverse (R) primers used for PCR were as follows: rat-GADPH-F, 5′-TAG GAG CCA GGG CAG TAA TCT-3′ and rat-GADPH-R, 5′-CGT TAC ATC CGT AAA GAC CTC-3′; rat-SIRT2-F, 5′-GGA GTC AAA ATC CAT GCC GC-3′ and rat-SIRT2-R, 5′-AAC CCT TCT TTG CCC TTG CT-3′, mouse-GADPH-F, 5′-CAA GGA GTA AGA AAC CCT GGA CC-3′ and mouse-GADPH-R, 5′-CGA GTT GGA TAG GGC CTC T-3′; mouse-SIRT2-F, 5′-TCA TCT GTT TGG GAG CC-3′ and mouse-SIRT2-R, 5′-TCC GTC TGG CCT GTC TTT TC-3′.

### Western blotting

Total protein was isolated as follows. First, the neurons were lysed using RIPA buffer containing NaF and protease inhibitors for 30 min on ice. Substantia nigra tissue obtained from mice was homogenized in ice-cold RIPA buffer containing NaF and protease inhibitors. The lysates were centrifuged at 12,000 × *g* for 15 min at 4 °C, and the supernatant fractions were collected. The supernatant fractions were re-suspended in 1× SDS sample buffer and boiled at 98 °C for 5 min.

Cell fractionation was conducted as follows (Protein Assay Kit, Beyotime Biotechnology): cells were collected in PBS and washed twice with cold PBS. The cell pellet was re-suspended in hypotonic buffer and incubated on ice for 15 min. Next, buffer was added to the cell suspension, and the sample was vortexed for 10 s. The homogenate was centrifuged for 5 min at 12,000 × *g* and 4 °C to obtain the cytoplasmic (supernatant) and nuclear (pellet) fractions. The nuclear pellet was re-suspended in complete cell extraction buffer and incubated on ice for 30 min, with shaking at 5-min intervals. The nuclear lysate was centrifuged at 12,000 × *g* and 4 °C for 15 min to obtain the nuclear fraction (supernatant). Then, 60–100 μg of protein was used for western blotting. Samples were separated on 10% SDS-PAGE gels and transferred to a PVDF membrane. The membranes were sequentially incubated with primary and secondary antibodies. Proteins were detected using horseradish peroxidase-coupled goat anti-mouse IgG or anti-rabbit IgG antibodies and an ECL western blotting detection system.

Soluble and insoluble proteins were extracted as follows. Substantia nigra tissues obtained from mice were homogenized in an ice-cold cocktail of 1% Tx-100 in Tris-buffered saline (TBS) (50 mM Tris, 150 mM NaCl, pH 7.4) containing protease and phosphatase protease inhibitors. Neurons were added to this cocktail at 4 °C. Lysates were centrifuged at 100,000 × *g* for 30 min, and the supernatant was harvested as a TX-soluble fraction. The TX-insoluble proteins were extracted using 2% (w/v) SDS/TBS and finally reconstituted in an equal volume of SDS/TBS buffer. Proteins were separated using SDS-PAGE. We confirmed all blots derive from the same experiment and were processed in parallel.

### Immunofluorescence staining

For cell staining, cells were grown on glass cover slides. After treatment, cells were fixed with 4% paraformaldehyde. For tissue staining, substantia nigra sections were used. Mouse brains were obtained after anesthetizing mice with sodium pentobarbital (40 mg/ml, i.p.) and performing transcardial perfusion with 0.9% NaCl. Subsequently, the brains were fixed in 4% paraformaldehyde dissolved in 0.1 M phosphate buffer at 4 °C. Fixed brains were transferred and stored in a 30% sucrose solution until they sank. Brains were then frozen and sectioned using a sliding microtome (Leica, Germany) into 20-μm coronal slices and collected in 0.01 M PBS. Slices were permeabilized in 0.3% Triton X-100 for 15 min and then blocked with 10% serum in PBS for 1 h. The sections were then incubated overnight with the primary antibodies at 4 °C. After three washes with PBS, the cells were incubated with secondary antibodies. Nuclei were stained using DAPI.

### Rotarod test

An accelerating rotarod (35 cm long and 6 cm in diameter) was suspended at a height of 29 and used to test the general sensorimotor coordination of the experimental mice. The mice were given 60 min to adjust to the new surroundings prior to the test. Each mouse was placed onto the center of the moving rod (40 rpm) and trained on this apparatus for 3 min. Subsequently, the speed of the rotarod was increased from 8 to 40 rpm over a 5-min period. The time at which each mouse fell from the rod was automatically recorded by the detector. The apparatus was cleaned with ethanol between each test. Mice underwent five trials a day for three consecutive days, with a 30-min interval between trials.

### Open field test

The exploratory and reactive behavior of mice in a novel space was tested in a Plexiglas cage (44 × 44 cm). Mice were individually placed at the center of a brightly lit open field to induce anxiety. Their movements were then tracked for the next 20 min. The spontaneous locomotory activity was measured using an activity monitor connected to a computer that recorded the mouse’s position every millisecond.

### RNA-Seq Analysis

Total RNA was extracted from approximately 2 × 10^7^ HEK293 cells using the TRIzol reagent (Takara). All the RNA samples were processed in the Beijing Genome Institute (BGI, Shenzhen, China), and library preparation, sequencing, and bioinformatics analysis were performed. Briefly, DEGs were identified based on the following two criteria: *(1) absolute fold-change >1.2 and (2) *P* value <0.05. DEG data were normalized by employing *Z*-score transformation, and the heatmap was generated using R project (gplots). Further, the GO and KEGG pathway enrichment of DEGs was analyzed using DAVID Bioinformatics Resources 6.7 (National Institute of Allergy and Infectious Diseases (NIAID), NIH, https://david.ncifcrf.gov)^41^ and the KEGG public database (Kanehisa Laboratories, http://www.kegg.jp)^42^ respectively, based on the number of unigene alignments within DEGs.

### Recombinant protein purification and in vitro kinase assay

Full-length human SIRT2 (WT, S331A, S335A, and S331AS335A mutant) was cloned into pGEX-4T3. The GST-tagged form of this protein was expressed in *Escherichia coli* BL21 cells after induction with 0.1 mM isopropyl β-d-thiogalactopyranoside (IPTG) for 12 h at 37 °C. The recombinant SIRT2 proteins were purified using glutathione sepharose beads. In vitro kinase reactions were conducted at 30 °C for 30 min in a reaction buffer containing 1–2 μg of the SIRT2 substrate and 20 μM ATP, with or without 25 ng of active Cdk5 kinase. The reaction was terminated by adding 1× SDS sample loading buffer. The reaction mixture was separated using SDS-PAGE, and the phosphorylation state was analyzed using western blotting with a phosphoserine/threonine antibody.

### Co-immunoprecipitation and GST pull-down assay

Primary cultured neurons were lysed in RIPA (weak) buffer containing protease inhibitors. Lysates were incubated with IgG or an anti-SIRT2 antibody (2 μg) at 4 °C overnight. Then, they were incubated with 50 μl of protein G plus/protein A agarose at 4 °C for 2 h. After three washes, the immune complexes were boiled for 5 min in 1× SDS sample buffer and analyzed using 10% SDS-PAGE. For the GST pull-down assay, lysates obtained from primary culture neurons were incubated with purified GST or GST-SIRT2 for 4 h at 4 °C. GST agarose beads were added to the mixture and incubated for 2 h at 4 °C. Immune complexes obtained were washed five times with wash buffer and subjected to western blot analysis.

### Peptides and viability assay

The two peptides used were Scramble-KTAPSAPKPPSS and SIRT2-TSASPKKSPPPA. Each of them was conjugated to myristic acid at their N-terminal. All peptides were purchased from KareBay Biochem, Inc. The peptides were 95% pure and kept at −20 °C. Primary culture neurons were pretreated with myr-SIRT2 or myr-Scramble and subsequently treated with 100 μM MPP^+^ for 48 h. The neuronal survival percentage was detected using an MTT assay.

### Statistical analysis

All data are presented as the mean ± SD and are representative of at least three independent experiments. For pairwise comparisons, unpaired *t-*tests were used. For comparisons of more than two treatment groups, one-way ANOVA or two-way ANOVA followed by Tukey’s post hoc tests were used. A *P* value less than 0.05 was considered statistically significant (*P < 0.05, **P < 0.01, and ***P < 0.001).

### Reporting summary

Further information on research design is available in the Nature Research Reporting Summary linked to this article.

## Supplementary information

Supplementary

Reporting Summary

## Data Availability

The data supporting the findings of this study are available within the article and Supplementary Information files. The data for this study are available from the corresponding author on reasonable request. The data that support the findings of this study have been deposited into CNGB Sequence Archive (CNSA) of China National GeneBank DataBase (CNGBdb) with accession number CNP0002710.

## References

[1] Malpartida, A. B., Williamson, M., Narendra, D. P., Wade-Martins, R. & Ryan, B. J. Mitochondrial dysfunction and mitophagy in Parkinson’s disease: from mechanism to therapy. *Trends Biochem. Sci.*10.1016/j.tibs.2020.11.007 (2020). 10.1016/j.tibs.2020.11.007 33323315

[2] Mazzocchi M, Collins LM, Sullivan AM, O’Keeffe GW (2020). The class II histone deacetylases as therapeutic targets for Parkinson’s disease. Neuronal Signal..

[3] Du G (2010). Drosophila histone deacetylase 6 protects dopaminergic neurons against {alpha}-synuclein toxicity by promoting inclusion formation. Mol. Biol. Cell.

[4] Wu, Q., Yang, X., Zhang, L., Zhang, Y. & Feng, L. Nuclear accumulation of histone deacetylase 4 (HDAC4) exerts neurotoxicity in models of Parkinson’s disease. *Mol. Neurobiol.*10.1007/s12035-016-0199-2 (2016). 10.1007/s12035-016-0199-2 27785754

[5] Choong CJ (2016). A novel histone deacetylase 1 and 2 isoform-specific inhibitor alleviates experimental Parkinson’s disease. Neurobiol. Aging.

[6] Pinho BR (2016). Pharmacological modulation of HDAC1 and HDAC6 in vivo in a zebrafish model: therapeutic implications for Parkinson’s disease. Pharmacol. Res..

[7] Yeong KY, Berdigaliyev N, Chang Y (2020). Sirtuins and their implications in neurodegenerative diseases from a drug discovery perspective. ACS Chem. Neurosci..

[8] Chandrasekaran K (2019). Overexpression of Sirtuin 1 protein in neurons prevents and reverses experimental diabetic neuropathy. Brain J. Neurol..

[9] Zhou ZD, Tan EK (2020). Oxidized nicotinamide adenine dinucleotide-dependent mitochondrial deacetylase sirtuin-3 as a potential therapeutic target of Parkinson’s disease. Ageing Res. Rev..

[10] Opitz, C. A. & Turcan, S. From anti-aging drugs to cancer therapy: is there a potential for sirtuin activators in gliomas? *Neuro-oncology*, 10.1093/neuonc/noaa234 (2020). 10.1093/neuonc/noaa234 PMC7849941 33059365

[11] Grabowska W, Sikora E, Bielak-Zmijewska A (2017). Sirtuins, a promising target in slowing down the ageing process. Biogerontology.

[12] Donmez G (2012). SIRT1 protects against alpha-synuclein aggregation by activating molecular chaperones. J. Neurosci..

[13] Wu Y (2011). Resveratrol-activated AMPK/SIRT1/autophagy in cellular models of Parkinson’s disease. Neuro-Signals.

[14] Harrison IF, Smith AD, Dexter DT (2018). Pathological histone acetylation in Parkinson’s disease: neuroprotection and inhibition of microglial activation through SIRT 2 inhibition. Neurosci. Lett..

[15] Eskandarian HA (2013). A role for SIRT2-dependent histone H3K18 deacetylation in bacterial infection. Science.

[16] North BJ, Verdin E (2007). Interphase nucleo-cytoplasmic shuttling and localization of SIRT2 during mitosis. PloS ONE.

[17] Gupta KK, Singh SK (2019). Cdk5: a main culprit in neurodegeneration. Int. J. Neurosci..

[18] Wang Q (2018). CDK5-mediated phosphorylation-dependent ubiquitination and degradation of E3 ubiquitin ligases GP78 accelerates neuronal death in Parkinson’s disease. Mol. Neurobiol..

[19] Cheng X (2020). The BRCC3 regulated by Cdk5 promotes the activation of neuronal NLRP3 inflammasome in Parkinson’s disease models. Biochem. Biophys. Res. Commun..

[20] Zhang Q (2018). Cdk5 suppression blocks SIRT1 degradation via the ubiquitin-proteasome pathway in Parkinson’s disease models. Biochim. Biophys. Acta Gen. Subj..

[21] Zhang P (2016). Cdk5-dependent activation of neuronal inflammasomes in Parkinson’s Disease. Mov. Disord..

[22] Wang C (2020). GPS 5.0: an update on the prediction of kinase-specific phosphorylation sites in proteins. Genomics Proteomics Bioinformatics.

[23] Obenauer JC, Cantley LC, Yaffe MB (2003). Scansite 2.0: proteome-wide prediction of cell signaling interactions using short sequence motifs. Nucleic Acids Res..

[24] Plotnikov A (2015). The nuclear translocation of ERK1/2 as an anticancer target. Nat. Commun..

[25] Dillin A, Kelly JW (2007). Medicine. The yin-yang of sirtuins. Science.

[26] Zhang Y, Anoopkumar-Dukie S, Arora D, Davey AK (2020). Review of the anti-inflammatory effect of SIRT1 and SIRT2 modulators on neurodegenerative diseases. Eur. J. Pharmacol..

[27] Yao YL, Yang WM (2011). Beyond histone and deacetylase: an overview of cytoplasmic histone deacetylases and their nonhistone substrates. J. Biomed. Biotechnol..

[28] Jiao FJ (2017). CDK5-mediated phosphorylation of XBP1s contributes to its nuclear translocation and activation in MPP(+)-induced Parkinson’s disease model. Sci. Rep..

[29] Nardozzi JD, Lott K, Cingolani G (2010). Phosphorylation meets nuclear import: a review. Cell Commun. Signal..

[30] Outeiro TF (2007). Sirtuin 2 inhibitors rescue alpha-synuclein-mediated toxicity in models of Parkinson’s disease. Science.

[31] Di Fruscia P (2015). The discovery of a highly selective 5,6,7,8-tetrahydrobenzo[4,5]thieno[2,3-d]pyrimidin-4(3H)-one SIRT2 inhibitor that is neuroprotective in an in vitro Parkinson’s disease model. ChemMedChem.

[32] Vaquero A (2006). SirT2 is a histone deacetylase with preference for histone H4 Lys 16 during mitosis. Genes Dev..

[33] Inoue T, Hiratsuka M, Osaki M, Oshimura M (2007). The molecular biology of mammalian SIRT proteins: SIRT2 in cell cycle regulation. Cell Cycle.

[34] Folch J (2012). Role of cell cycle re-entry in neurons: a common apoptotic mechanism of neuronal cell death. Neurotox. Res..

[35] Smeyne M, Smeyne RJ (2013). Glutathione metabolism and Parkinson’s disease. Free Radic. Biol. Med..

[36] Tufi R (2014). Enhancing nucleotide metabolism protects against mitochondrial dysfunction and neurodegeneration in a PINK1 model of Parkinson’s disease. Nat. Cell Biol..

[37] Meles SK (2020). Abnormal pattern of brain glucose metabolism in Parkinson’s disease: replication in three European cohorts. Eur. J. Nucl. Med. Mol. imaging.

[38] Drosten M, Barbacid M (2020). Targeting the MAPK pathway in KRAS-driven tumors. Cancer Cell.

[39] Nie H (2011). Silencing of SIRT2 induces cell death and a decrease in the intracellular ATP level of PC12 cells. Int. J. Physiol. Pathophysiol. Pharmacol..

[40] Shu Y (2016). Parkinson-related LRRK2 mutation R1628P enables Cdk5 phosphorylation of LRRK2 and upregulates its kinase activity. PloS ONE.

[41] Huang da W, Sherman BT, Lempicki RA (2009). Systematic and integrative analysis of large gene lists using DAVID bioinformatics resources. Nat. Protoc..

[42] Kanehisa M, Sato Y, Furumichi M, Morishima K, Tanabe M (2019). New approach for understanding genome variations in KEGG. Nucleic Acids Res..

