# Supplementary material for: Cdk5 phosphorylation-induced SIRT2 nuclear translocation promotes the death of dopaminergic neurons in Parkinson’s disease
Source: NPJ Parkinsons Dis. 2022 Apr 20;8:46. doi: 10.1038/s41531-022-00311-0 (PMC9021196; doi:10.1038/s41531-022-00311-0)
Supplement: Supplementary file 1 — Supplementary [file 41531_2022_311_MOESM1_ESM.pdf]

Supplementary information

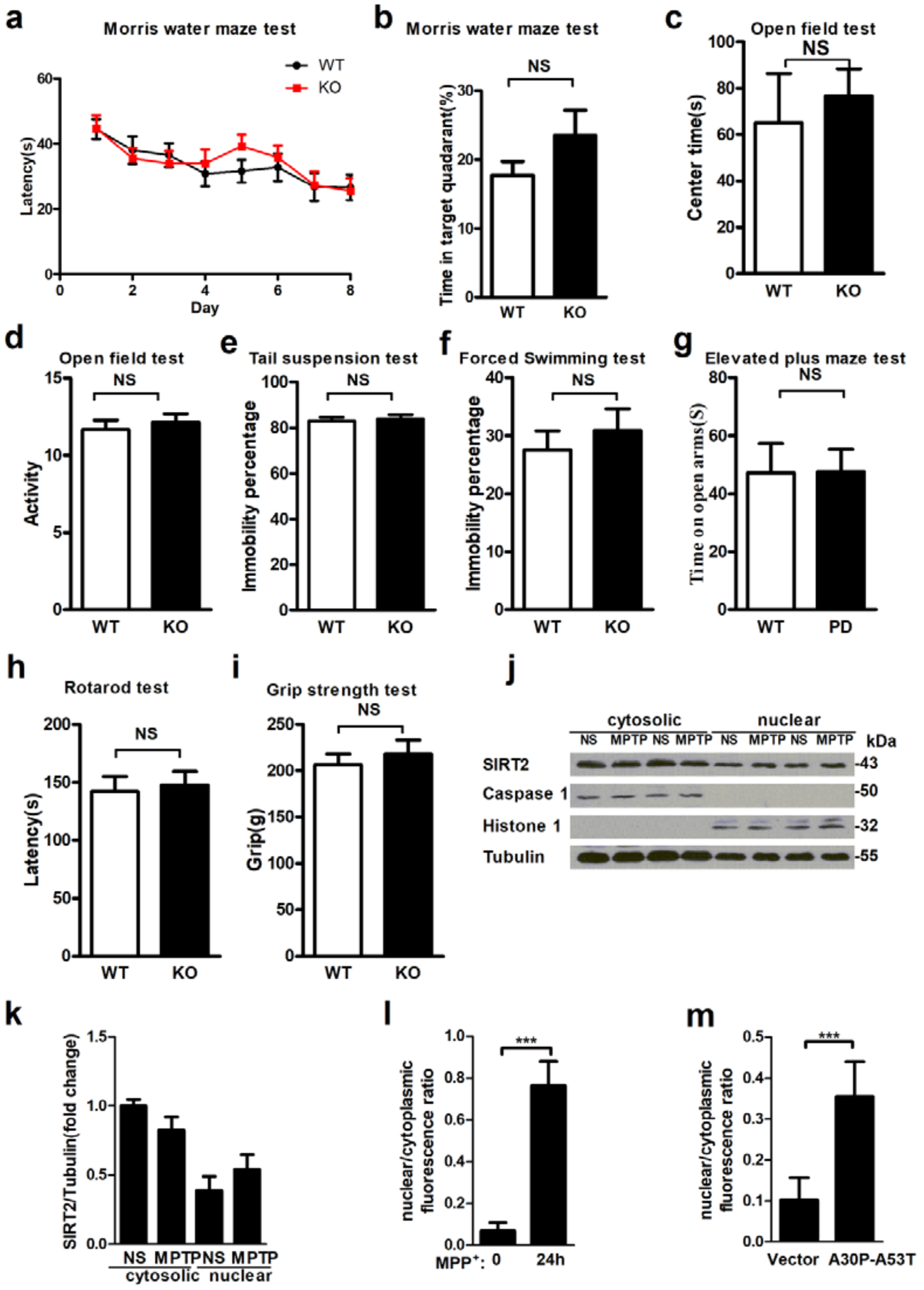

Supplementary Figure 1. Behavioral tests in SIRT2 KO mice. Learning and memory were evaluated using the Morris water maze test (a and b) in SIRT2 KO mice and WT controls at 6 months of age (n=15). Anxiety-related behavior was examined using the open field test (c and d), tail suspension test (e), forced swim test (f), and

elevated plus maze test (g) in SIRT2 KO mice and WT controls (n=15). Motor function was evaluated using the rotarod test (h) and grip strength test (i) in SIRT2 KO mice and WT controls (n=15). (j) The localization of SIRT2 was detected using nuclear/cytosolic immunoblotting in the SNpc of mice treated with NS or MPTP. (k) Quantification of SIRT2 protein levels in the cytoplasm and nucleus (separate from [j]). (l) Nuclear localization of SIRT2 was quantitatively analyzed in primary culture neurons treated with 50 μM MPP^+ for 24 h (Fig. 3*c*) based on the ratio of nuclear to cytoplasmic fluorescence. Fluorescence intensity was detected using Image J software, and 15 stained cells were analyzed. (m) Nuclear localization of SIRT2 was quantitatively analyzed in SH-SY5Y cells transiently transfected with a GFP vector or GFP-α-synuclein-A30P*A53T plasmid (Fig. 3*f*) based on the ratio of nuclear to cytoplasmic fluorescence. Fluorescence intensity was detected using Image J software, and 15 stained cells were analyzed. All data are presented as the means ± SD. Statistical analyses were conducted using two-way ANOVA followed by Tukey's post-hoc test in a and k. Statistical analyses were conducted using an unpaired t-test in b-i and l-M. *** $P < 0.001$.

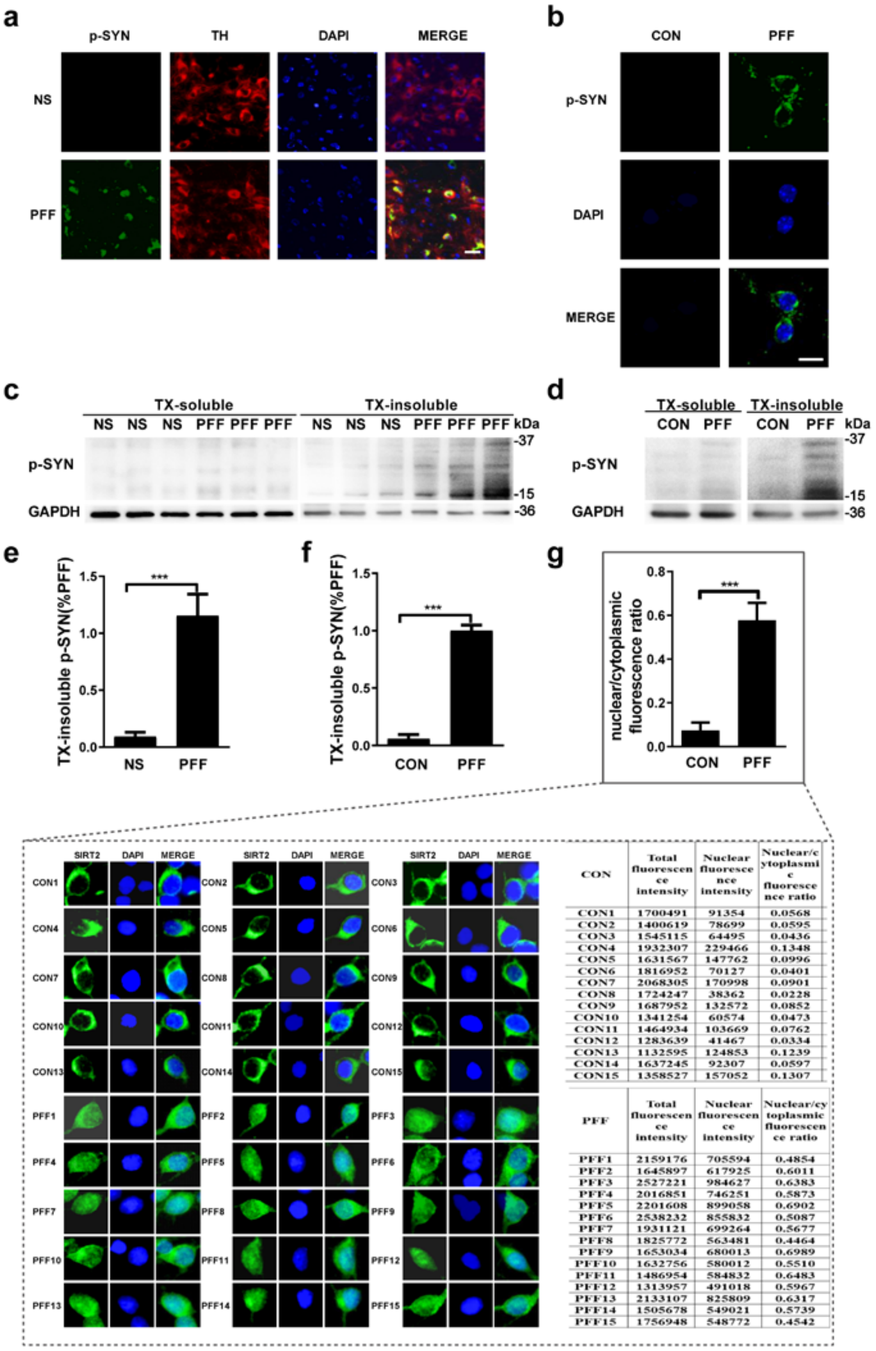

CON	Total fluorescence intensity	Nuclear fluorescence intensity	Nuclear/cytoplasmic fluorescence ratio
CON1	1700491	91354	0.0568
CON2	1400619	78699	0.0595
CON3	1545115	64495	0.0436
CON4	1932307	229466	0.1348
CON5	1631567	147762	0.0996
CON6	1816952	70127	0.0401
CON7	2068305	170998	0.0901
CON8	1724247	38362	0.0228
CON9	1687952	132572	0.0852
CON10	1341254	60574	0.0473
CON11	1464934	103669	0.0762
CON12	1283639	41467	0.0334
CON13	1132595	124853	0.1239
CON14	1637245	92307	0.0597
CON15	1358527	157052	0.1307

PFF	Total fluorescence intensity	Nuclear fluorescence intensity	Nuclear/cytoplasmic fluorescence ratio
PFF1	2159176	705594	0.4854
PFF2	1645897	617925	0.6011
PFF3	2527221	984627	0.6383
PFF4	2016851	746251	0.5873
PFF5	2201608	899058	0.6902
PFF6	2538232	855832	0.5087
PFF7	1931121	699264	0.5677
PFF8	1825772	563481	0.4464
PFF9	1653034	680013	0.6989
PFF10	1632756	580012	0.5510
PFF11	1486954	584832	0.6483
PFF12	1313957	491018	0.5967
PFF13	2133107	825809	0.6317
PFF14	1505678	549021	0.5739
PFF15	1756948	548772	0.4542

Supplementary Figure 2. Pathological manifestations in the α-synuclein preformed fibril (PFF) PD model. (a) α-synuclein Ser129 phosphorylation in the SNpc in mice assessed 35 d after the striatal stereotactic injection of 2 μl (per side) normal saline (NS) or α-synuclein PFF (Abcam, ab246002). Representative images of

TH (red) and p-Ser129 α-synuclein (green) immunostaining in the SNpc are shown. The scale bar represents 20 μm. (b) α-synuclein Ser129 phosphorylation in primary culture neurons treated with 4 μg/ml PFF for 7 d. Representative images of p-Ser129 α-synuclein (green) immunostaining are shown. The scale bar represents 20 μm. (c) Mice were treated with normal saline (NS) or α-synuclein PFF for 35 d. Proteins were sequentially extracted from the SNpc using 1% Tx-100 (soluble protein) and 2% SDS (insoluble protein). Western blotting was used to analyze the level of p-α-synuclein in the lysates. (d) Primary culture neurons were treated with 4 μg/ml PFF, and 7 d later, proteins were sequentially extracted using 1% Tx-100 (soluble protein) and 2% SDS (insoluble protein). Western blotting was used to analyze the level of p-α-synuclein in the lysates. (e) Bar graph showing the protein levels of insoluble p-α-synuclein in the insoluble fraction (separate from [c]). (f) Bar graph showing the protein levels of insoluble p-α-synuclein in the insoluble fraction (separate from [d]). (g) Nuclear localization of SIRT2 was quantitatively analyzed in primary culture neurons treated with 4 μg/ml PFF for 7 d (Fig. 3*h*) based on the ratio of nuclear to cytoplasmic fluorescence. Fluorescence intensity was detected using Image J software, and 15 stained cells were analyzed. All data are presented as the means ± SD. Statistical analyses were conducted using an unpaired t-test in e, f and g. *** $P < 0.001$.

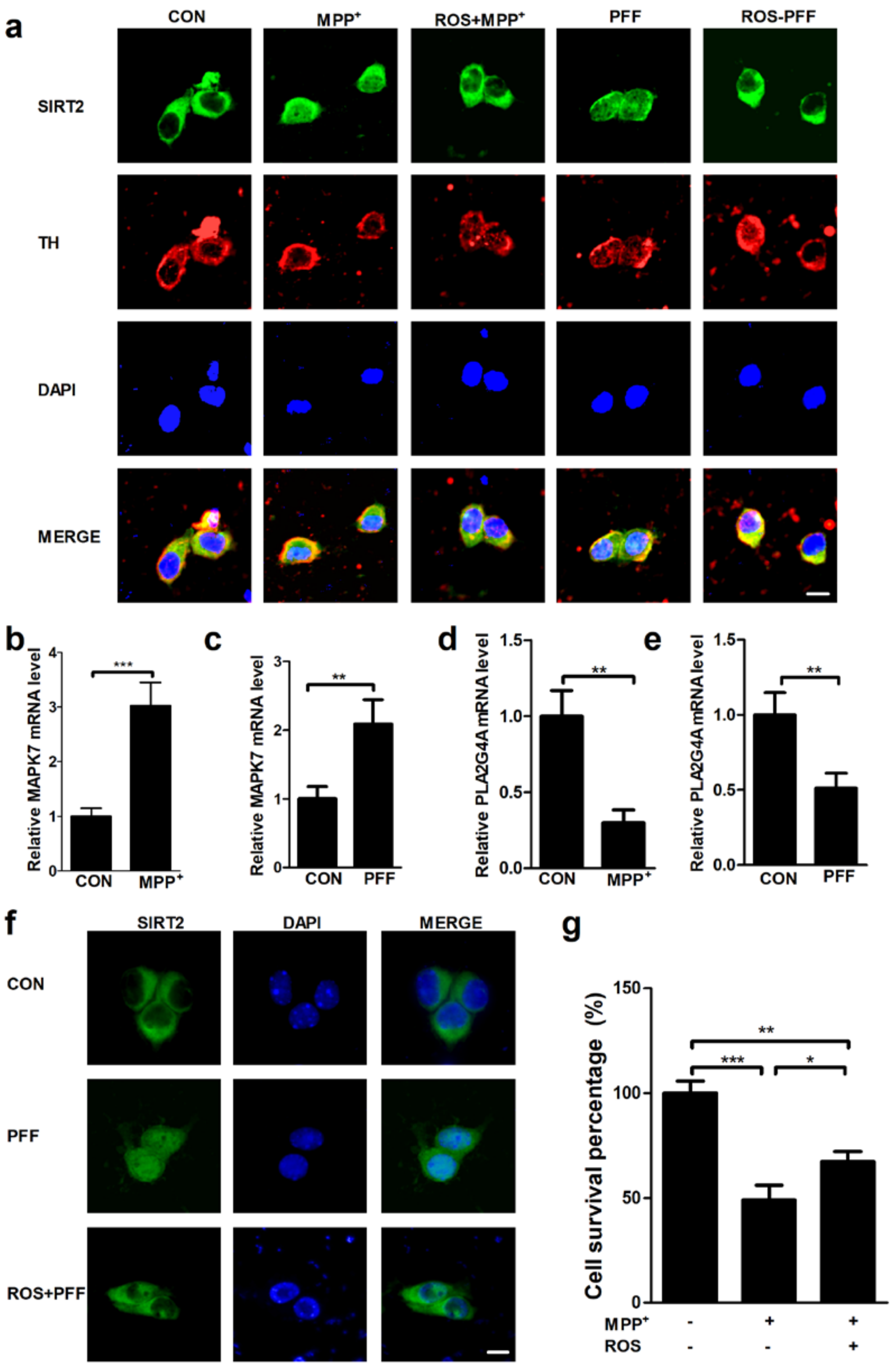

Supplementary Figure 3. Cdk5 is involved in the nuclear translocation of SIRT2 in cellular PD model of midbrain dopaminergic neurons. (a) Cultured midbrain dopaminergic neurons were pretreated with 10 μM roscovitine (ROS) for 0.5 h and

subsequently treated with 50 μM MPP^+ for 24 h or treated with 4 μg/ml PFF alone for 7 d. The cytoplasmic and nuclear distribution of SIRT2 was detected using immunofluorescence. The scale bar represents 10 μm. (b) mRNA levels of MAPK7 in primary culture neurons treated with 50 μM MPP^+ for 24 h were assessed using qPCR (n=3). (c) mRNA levels of MAPK7 in primary culture neurons treated with 4 μg/ml PFF for 7 d were assessed using qPCR (n=3). (d) mRNA levels of PLA2G4A in primary culture neurons treated with 50 μM MPP^+ for 24 h were assessed using qPCR (n=3). (e) mRNA levels of PLA2G4A in primary culture neurons treated with 4 μg/ml PFF for 7 d were assessed using qPCR (n=3). (f) Localization of SIRT2 was detected using nuclear/cytoplasmic immunofluorescence staining in primary culture neurons treated with 4 μg/ml PFF for 7 d with or without pre-treatment with 10 μM roscovitine (ROS) for 0.5 h. The scale bar represents 10 μm. (g) Primary culture neurons were pretreated with 10 μM ROS for 0.5 h and subsequently treated with 100 μM MPP^+ for 48 h. The survival percentage of neurons was detected using the MTT assay (n=3). All data are presented as the means ± SD. Statistical analyses were conducted using an unpaired t-test in b, c, d and e. Statistical analyses were conducted using one-way ANOVA followed by Tukey's post-hoc test in g.

* $P < 0.05$, ** $P < 0.01$, *** $P < 0.001$.

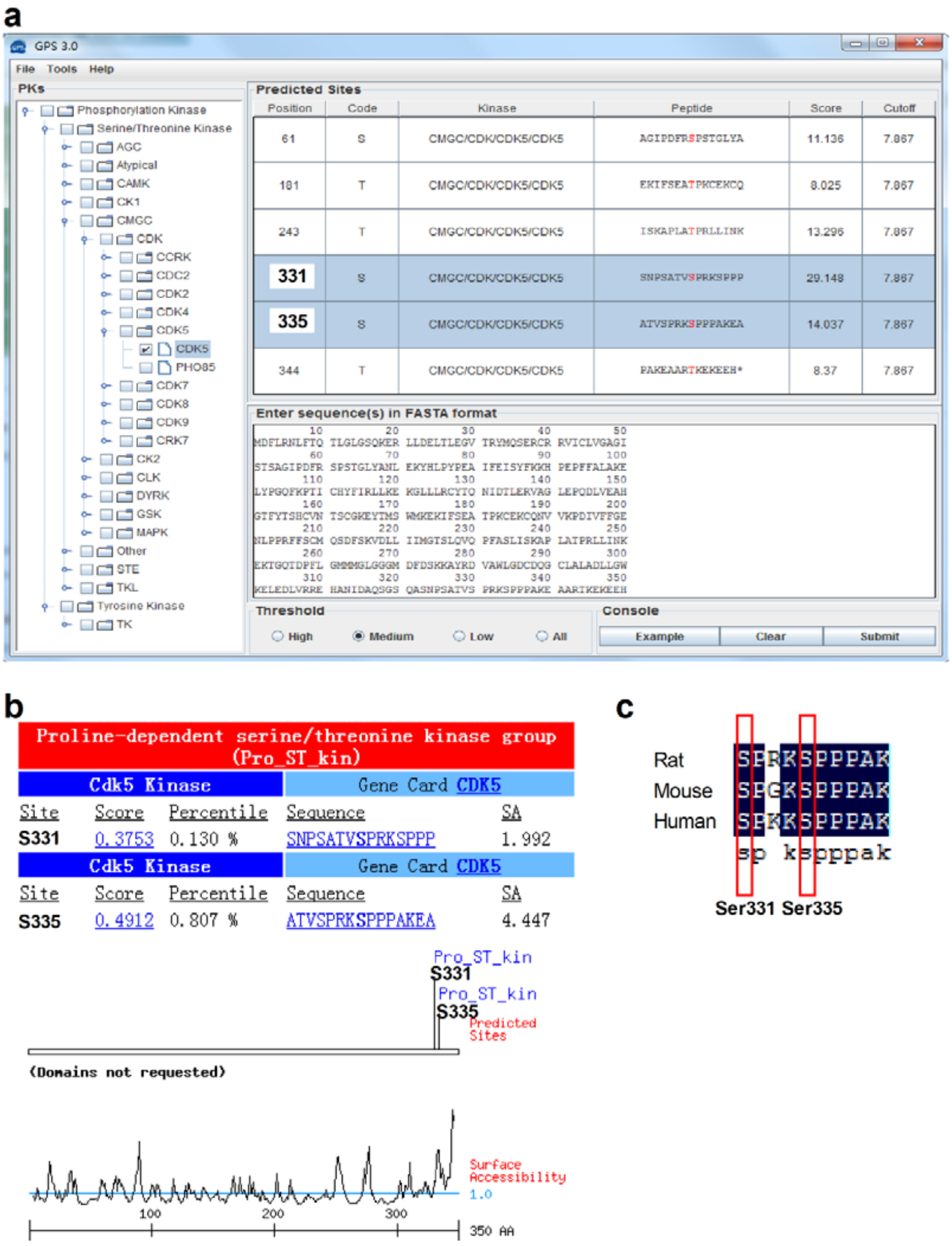

Supplementary Figure 4. Bioinformatics analysis of Cdk5-dependent SIRT2 phosphorylation. (a) Computational prediction of Cdk5-dependent phosphorylation sites in the SIRT2 protein using GPS 3.0 (Group-based Prediction System, version 3.0). (b) Scansite 3beta was used to identify motifs and amino acid sites within the SIRT2 protein that are likely to be phosphorylated by specific protein kinases like

Cdk5. (c) SIRT2 protein sequences from different species were compared with multiple sequence alignment using DNAMAN software.

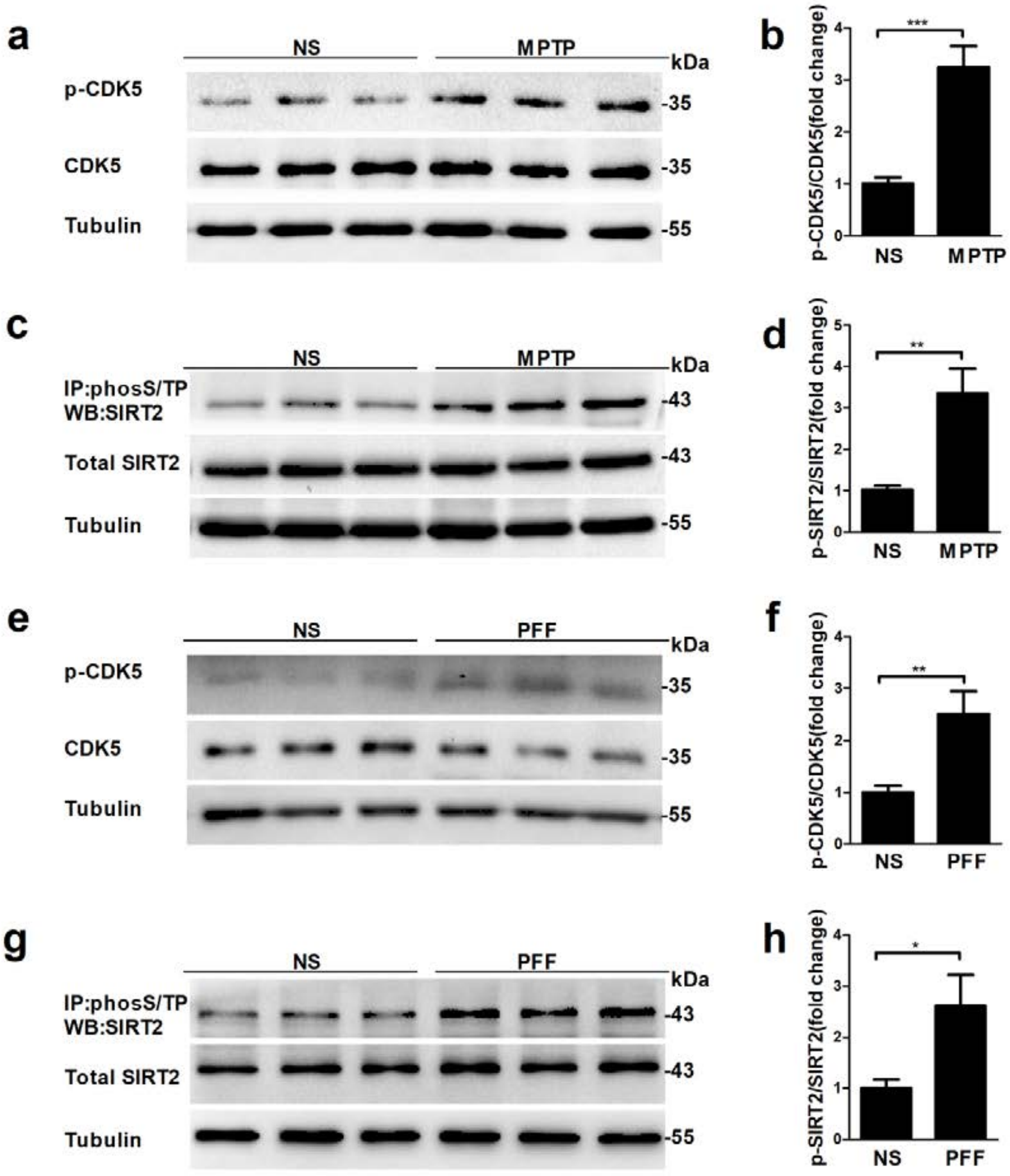

Supplementary Figure 5. CDK5 and SIRT2 phosphorylation was induced by MPTP and PFF in the mouse SNpc. (a) CDK5 phosphorylation levels in the SNpc were measured in mice treated with normal saline (NS) or MPTP using western blot. (b) Quantification of p-CDK5/CDK5 protein levels. (c) SIRT2 phosphorylation levels in the SNpc were measured in mice treated with normal saline or MPTP using

immunoprecipitation. (d) Quantification of p-SIRT2/SIRT2 protein levels. (e) CDK5 phosphorylation levels in the SNpc were measured in mice treated with normal saline or PFF using western blot. (f) Quantification of p-CDK5/CDK5 protein levels. (g) SIRT2 phosphorylation levels in the SNpc were measured in mice treated with normal saline or PFF using immunoprecipitation. (h) Quantification of p-SIRT2/SIRT2 protein levels. All data are presented as the means ± SD. Statistical analyses were conducted using an unpaired t-test. * $P < 0.05$, ** $P < 0.01$, *** $P < 0.001$.

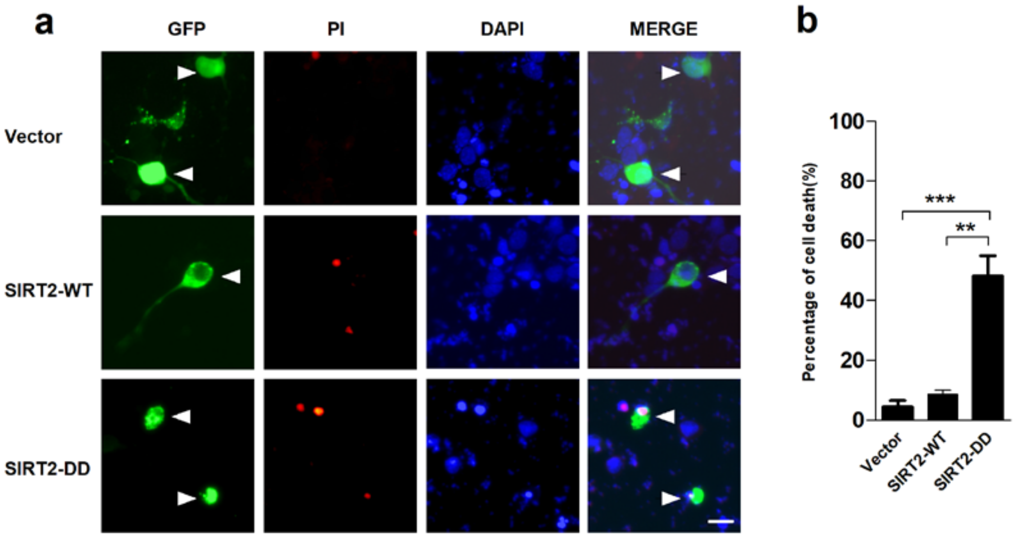

Supplementary Figure 6. SIRT2 mutation DD (double mutation at S331D and S335D) promotes neuronal death. (a) Primary culture neurons were transiently transfected with GFP vector, GFP-SIRT2 WT, or GFP-SIRT2 DD plasmids, and subsequently stained for GFP (green), PI (red), and DAPI (blue). The scale bar represents 20 μm. (b) GFP-positive cells (green) that were also PI-positive (red) were considered to be dead neurons. The percentage of PI-positive cells among at least 100 GFP-positive cells was measured for each group (n=3). All data are presented as the

means ± SD. Statistical analyses were conducted using one-way ANOVA followed by Tukey's post-hoc test. $^{**}P < 0.01$, $^{***}P < 0.001$.

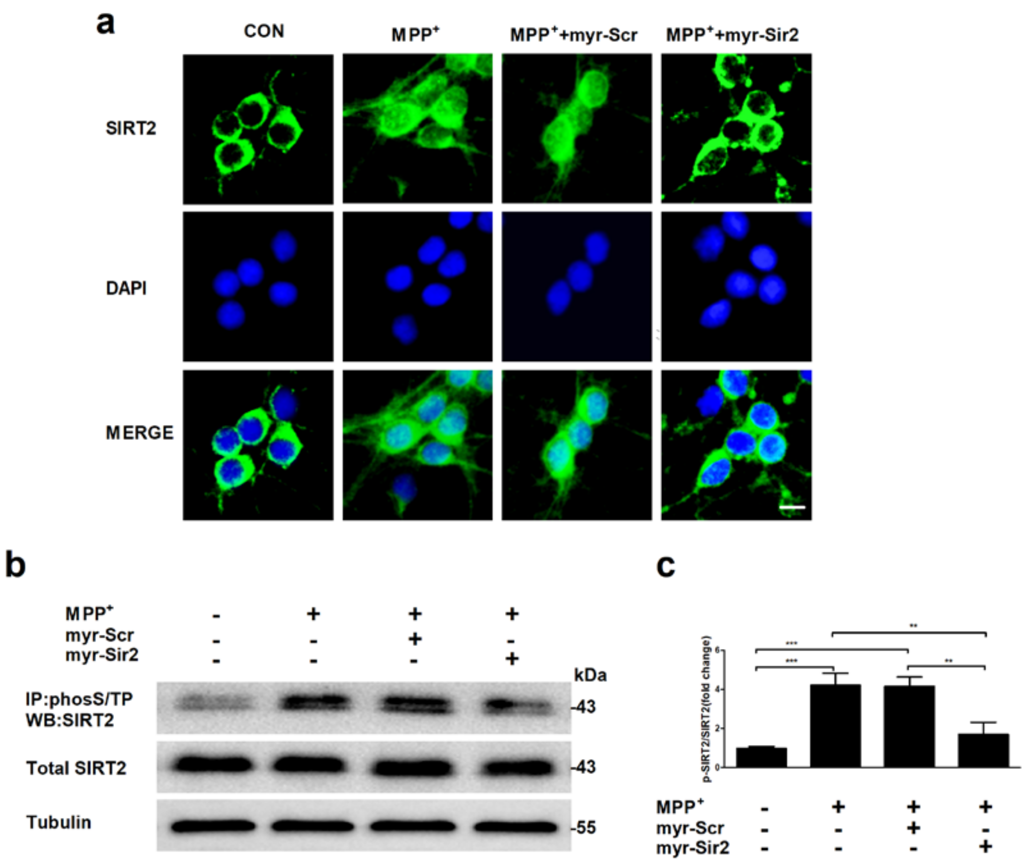

Supplementary Figure 7. Myr-SIRT2$_{328\text{-}339}$ interference peptide rescues the nuclear translocation and phosphorylation of SIRT2. (a) Primary culture neurons were pretreated with the SIRT2$_{328\text{-}339}$ peptide or a Scramble peptide conjugated to myristic acid (Myr) and subsequently treated with 50 μM MPP^+ for 24 h. The localization of SIRT2 was detected using nuclear/cytosolic immunofluorescence. The scale bar represents 10 μm. (b) SIRT2 phosphorylation was detected using immunoprecipitation. (c) Quantification of p-SIRT2/SIRT2 protein levels. All data are presented as the means ± SD. Statistical analyses were conducted using one-way ANOVA followed by Tukey's post-hoc test. $^{**}P < 0.01$, $^{***}P < 0.001$.

Supplementary Methods

Antibodies and chemical reagents. The rabbit polyclonal anti-Sirt2 (s8447) antibody was purchased from Sigma-Aldrich. The rabbit polyclonal anti-phosS/TP antibody (ab9344) and rabbit anti-Alpha-synuclein (S129) antibody (ab51253) were purchased from Abcam. The anti-CDK5 (sc-6247) and anti-p-CDK5 (sc-12919) antibodies were obtained from Santa Cruz Biotechnology. The rabbit anti -GAPDH polyclonal antibody (10842-1-AP) and rabbit anti-alpha synuclein polyclonal antibody (10494-1-AP) were purchased from Proteintech Group. The recombinant mouse alpha-synuclein protein preformed fibril (Active) (PFF, ab246002) was from Abcam.

Primary midbrain neuron culture. Dissociated midbrain neurons were cultured from rat embryos at embryonic day 16-18 on 6-well plates coated with poly-L-lysine (0.1 mg/ml). Neurons were fed with Neurobasal medium supplemented with 2% B27 (Invitrogen). After 24 h of plating, the cell division inhibitor AraC was added to the culture media at a final concentration of 10 μM. All treatments were performed at 7 days after plating.

qPCR. Total cellular RNA was extracted using the TRIZOL reagent (Thermo Fisher). RNA was reverse transcribed to cDNA using a qRT-PCR kit (Thermo Fisher) according to manufacturer's instructions. The PCR cycling conditions were as follows: 40 cycles with pre-denaturation at 95°C for 15 min, denaturation at 95°C for 10 s, and annealing and extension at 60°C for 30 s.

The qPCR primers were as follows: MAPK7-F: CGGGGCCTCAAATACATGCACTC, MAPK7-R: ACGGGCCATTCCAAAGTCACCAA; PLA2G4A-F: TTTGGGAGTTTCTGGCTCAC, PLA2G4A-R: TCTTCATTCTCGGTGCCTTT; β-actin-F: ACATCCGTAAAGACCTCTATGCC, β-actin-R: TACTCCTGCTTGCTGATCCAC. All results were normalized using internal controls, and fold changes were calculated based on relative quantification ($2^{-\Delta\Delta Ct}$).

Immunoprecipitation.

The phosS/TP antibodies were incubated with tissue lysates obtained using RIPA at 4°C overnight with gentle rotation. After incubation, protein A/G-Sepharose beads (Santa Cruz Biotechnology) were added to the lysates, and the mixture was rotated gently at 4°C for 1 h. Beads were precipitated after 10 min of stewing at 4°C. The beads were then washed thrice using cold RIPA buffer and resuspended in loading buffer (2×). After boiling and centrifugation, the supernatants were collected and tested using western blots.

Morris water maze (MWM) test. The MWM test, which is commonly used to examine learning and memory in rodents, was performed for behavioral assessments in SIRT2 KO mice. Briefly, the water maze (120-cm diameter) was filled with water mixed with non-toxic milk and divided into four quadrants: northeast (NE), southeast (SE), southwest (SW), and northwest (NW). The circular platform (10 cm in diameter) was located in the center of the NE quadrant and was submerged 2 cm below the surface. Each mouse was trained daily for 7 days (4 trials per day) to find the hidden

platform. After finding the platform, the mice were required to stay on it for 30 s. If they failed to reach the platform within 60 s, they were guided to the platform and allowed to remain there for the same period of time. The platform was removed after training, and each mouse was allowed 60 s for the probe trial. Learning was assessed by measuring the time required to find the platform. Data were collected using a digital camera, which was mounted above the pool and connected to a video recorder with an automated tracking system (China Daheng Group, Beijing, China). To characterize memory, the number of potential platform crossings and time spent in the target quadrant during the probe trial were assessed.

Tail suspension test. The tail suspension test is a behavioral assay in which rodents are placed in an inescapable stressful condition and hung by their tails. Briefly, mice were suspended using bands and hung from a hook mounted 50 cm above the floor for 5 min. The total duration for which the mice were immobile during the 5-min test period was measured. Immobility was defined as the lack of all movement, except for whisker movement and respiration.

Forced swim test. In the forced swim test, mice were forced to swim in a beaker filled with water (25 ± 1°C) and not allowed to escape. The total time spent immobile during the 6-min test period was observed. The experiment was video-recorded and analyzed post-recording.

Elevated plus maze test. The plus maze consisted of two open (25 × 5 × 0.5 cm) and two closed arms (25× 5 × 15 cm). It was kept at a height of 25 cm from floor, and a 60 W bulb was placed above it at a height of 100 cm. Each mouse was placed on the

central platform (5 cm × 5 cm) facing an open arm and allowed to explore the maze for 5 min. The amount of time spent in the open and closed arms was then calculated. The maze was cleaned with diluted alcohol between subsequent test sessions to eliminate any residual odor.

Grip strength test. The forelimb grip strength test was used to evaluate muscle strength in mice. The mice were held up, gripping a pull wire with only their front paws. The pull bar was steadily pulled back until the mice could no longer hold on to the wire. This test was repeated five times per mouse for each grip, and the highest force measurement was recorded.
