# Supplementary material for: Cdk5 phosphorylation-induced SIRT2 nuclear translocation promotes the death of dopaminergic neurons in Parkinson’s disease
Source: NPJ Parkinsons Dis. 2022 Apr 20;8:46. doi: 10.1038/s41531-022-00311-0 (PMC9021196; doi:10.1038/s41531-022-00311-0)
Supplement: Supplementary file 2 — Reporting Summary [file 41531_2022_311_MOESM2_ESM.pdf]

nature portfolio

Corresponding author(s): Yali Zhou
Bo Tian

Last updated by author(s): Mar 3, 2022

Reporting Summary

Nature Portfolio wishes to improve the reproducibility of the work that we publish. This form provides structure for consistency and transparency in reporting. For further information on Nature Portfolio policies, see our Editorial Policies and the Editorial Policy Checklist.

Statistics

For all statistical analyses, confirm that the following items are present in the figure legend, table legend, main text, or Methods section.

n/a	Confirmed	
☐	☒	The exact sample size (*n*) for each experimental group/condition, given as a discrete number and unit of measurement
☐	☒	A statement on whether measurements were taken from distinct samples or whether the same sample was measured repeatedly
☐	☒	The statistical test(s) used AND whether they are one- or two-sided<br>*Only common tests should be described solely by name; describe more complex techniques in the Methods section.*
☒	☐	A description of all covariates tested
☐	☒	A description of any assumptions or corrections, such as tests of normality and adjustment for multiple comparisons
☐	☒	A full description of the statistical parameters including central tendency (e.g. means) or other basic estimates (e.g. regression coefficient) AND variation (e.g. standard deviation) or associated estimates of uncertainty (e.g. confidence intervals)
☐	☒	For null hypothesis testing, the test statistic (e.g. *F*, *t*, *r*) with confidence intervals, effect sizes, degrees of freedom and *P* value noted<br>*Give P values as exact values whenever suitable.*
☒	☐	For Bayesian analysis, information on the choice of priors and Markov chain Monte Carlo settings
☒	☐	For hierarchical and complex designs, identification of the appropriate level for tests and full reporting of outcomes
☒	☐	Estimates of effect sizes (e.g. Cohen's *d*, Pearson's *r*), indicating how they were calculated

Our web collection on statistics for biologists contains articles on many of the points above.

Software and code

Policy information about availability of computer code

Data collection	No new softwares. Published softwares are listed with references in Methods.
Data analysis	Data analysis was described in the "Statistical analysis" section in the Methods.

For manuscripts utilizing custom algorithms or software that are central to the research but not yet described in published literature, software must be made available to editors and reviewers. We strongly encourage code deposition in a community repository (e.g. GitHub). See the Nature Portfolio guidelines for submitting code & software for further information.

Data

Policy information about availability of data

All manuscripts must include a data availability statement. This statement should provide the following information, where applicable:

- Accession codes, unique identifiers, or web links for publicly available datasets
- A description of any restrictions on data availability
- For clinical datasets or third party data, please ensure that the statement adheres to our policy

All data supporting the findings of this study are available within the figures of manuscript and its Supplementary Information files. The data that support the findings of this study have been deposited into CNGB Sequence Archive (CNSA) of China National GeneBank DataBase (CNGBdb) with accession number CNP0002710.

Field-specific reporting

Please select the one below that is the best fit for your research. If you are not sure, read the appropriate sections before making your selection.

☒ Life sciences ☐ Behavioural & social sciences ☐ Ecological, evolutionary & environmental sciences

For a reference copy of the document with all sections, see nature.com/documents/nr-reporting-summary-flat.pdf

Life sciences study design

All studies must disclose on these points even when the disclosure is negative.

Sample size	Sample size was determined based on the numbers reported in the field.
Data exclusions	No samples or animals were excluded from the analyses.
Replication	All data presented reflect findings that were reproducible.
Randomization	The mice were randomized to put into separate groups
Blinding	The investigators were blinded to the treatment of mice

Reporting for specific materials, systems and methods

We require information from authors about some types of materials, experimental systems and methods used in many studies. Here, indicate whether each material, system or method listed is relevant to your study. If you are not sure if a list item applies to your research, read the appropriate section before selecting a response.

Materials & experimental systems

n/a	Involved in the study	
☐	☒	Antibodies
☐	☒	Eukaryotic cell lines
☒	☐	Palaeontology and archaeology
☐	☒	Animals and other organisms
☒	☐	Human research participants
☒	☐	Clinical data
☒	☐	Dual use research of concern

Methods

n/a	Involved in the study	
☒	☐	ChIP-seq
☒	☐	Flow cytometry
☒	☐	MRI-based neuroimaging

Antibodies

Antibodies used	Antibody were purchased from standard commercial sources and the catalog numbers were provided in the Methods.
Validation	All the commerial antibodies were validated in reference to manufacturer's website or relevant citations.

Eukaryotic cell lines

Policy information about cell lines

Cell line source(s)	SY5Y cells ,HEK293 cells
Authentication	N/A
Mycoplasma contamination	NO
Commonly misidentified lines (See ICLAC register)	N/A

Animals and other organisms

Policy information about studies involving animals; ARRIVE guidelines recommended for reporting animal research

Laboratory animals	Sirt2 KO (Sirt2 KO, stock number: 012772), Cdk5 KO (Cdk5-loxP, stock number: 014156), and Tg-SNCA(Tg-SNCA, stock number: 008239) mice were purchased from Jackson Laboratories. The mice were given access to food and water ad libitum. The mice were

	group-housed under a 12 h light-dark cycle (lights on at 8:00 a.m.), at consistent humidity (50 ± 5 %), and ambient temperature (22 ± 1 °C).
Wild animals	Not applicable.
Field-collected samples	Not applicable.
Ethics oversight	All animal experiments were performed according to protocols approved by the Institutional Animal Ethics Committee

Note that full information on the approval of the study protocol must also be provided in the manuscript.
